# Broad-spectrum CRISPR-mediated inhibition of SARS-CoV-2 variants and endemic coronaviruses in vitro

**DOI:** 10.1038/s41467-022-30546-7

**Published:** 2022-05-19

**Authors:** Leiping Zeng, Yanxia Liu, Xammy Huu Wrynla, Timothy R. Abbott, Mengting Han, Yanyu Zhu, Augustine Chemparathy, Xueqiu Lin, Xinyi Chen, Haifeng Wang, Draven A. Rane, Jordan M. Spatz, Saket Jain, Arjun Rustagi, Benjamin Pinsky, Adrianna E. Zepeda, Anastasia P. Kadina, John A. Walker, Kevin Holden, Nigel Temperton, Jennifer R. Cochran, Annelise E. Barron, Michael D. Connolly, Catherine A. Blish, David B. Lewis, Sarah A. Stanley, Marie F. La Russa, Lei S. Qi

**Affiliations:** 1 Department of Bioengineering, Stanford University, Stanford, CA 94305 USA; 2 Department of Molecular and Cellular Biology, University of California, Berkeley, CA 94720 USA; 3 Department of Pediatrics, Stanford University, Stanford, CA 94305 USA; 4 University of California San Francisco, San Francisco, CA 94143 USA; 5 Department of Medicine, Stanford University, Stanford, CA 94305 USA; 6 Department of Pathology, Stanford University, Stanford, CA USA; 7 Synthego Corporation, Redwood City, CA USA; 8 Viral Pseudotype Unit, Medway School of Pharmacy, Chatham, Kent ME4 4TB, UK; 9 Lawrence Berkeley National Laboratory, Berkeley, CA 94720 USA; 10 Chan Zuckerberg BioHub, San Francisco, CA 94158 USA; 11 Sarafan ChEM-H, Stanford University, Stanford, CA 94305 USA

**Keywords:** SARS-CoV-2, Synthetic biology, CRISPR-Cas systems, Synthetic biology

## Abstract

A major challenge in coronavirus vaccination and treatment is to counteract rapid viral evolution and mutations. Here we demonstrate that CRISPR-Cas13d offers a broad-spectrum antiviral (BSA) to inhibit many SARS-CoV-2 variants and diverse human coronavirus strains with >99% reduction of the viral titer. We show that Cas13d-mediated coronavirus inhibition is dependent on the crRNA cellular spatial colocalization with Cas13d and target viral RNA. Cas13d can significantly enhance the therapeutic effects of diverse small molecule drugs against coronaviruses for prophylaxis or treatment purposes, and the best combination reduced viral titer by over four orders of magnitude. Using lipid nanoparticle-mediated RNA delivery, we demonstrate that the Cas13d system can effectively treat infection from multiple variants of coronavirus, including Omicron SARS-CoV-2, in human primary airway epithelium air-liquid interface (ALI) cultures. Our study establishes CRISPR-Cas13 as a BSA which is highly complementary to existing vaccination and antiviral treatment strategies.

## Abstract (web summary)

A major challenge in coronavirus vaccination and treatment is to counteract rapid viral evolution and mutations. Here the authors show that CRISPR-Cas13d can be used as a broad-spectrum antiviral to inhibit human coronaviruses, including new SARS-CoV-2 variants, combined with small molecule drugs for an enhanced antiviral effect in human primary cells.

## Introduction

Since 1918, over 11 human viral pandemics have been caused by RNA viruses, 8 of which used human-to-human respiratory spread as the major mode of transmission^1^. Three of these were due to beta-coronaviruses, including the ongoing COVID-19/SARS-CoV-2 pandemic that emerged in December 2019^2^. Despite intensive research, only a few antiviral drugs have been developed for wide clinical use to prevent or treat infection, e.g., inhibitors of the Influenza A virus (IAV) neuraminidase^3^ or inhibitors targeting the RNA-dependent RNA polymerase (RdRp) such as remdesivir^4^. This limited antiviral drug pipeline highlights a major unmet need for more facile antiviral drug development that can include diverse target mechanisms^5^.

The error-prone nature of the RdRp used by coronaviruses, IAV, and other RNA genome viruses generates a relatively high frequency of genetic variants with nucleotide changes that upon translation may result in amino acid substitutions^6^. During epidemic or pandemic outbreaks, newly arising viral variants may become predominant because of amino acid substitutions that increase viral infectiousness and human-to-human respiratory transmission and/or that help evade pre-existing immune responses, such as those mediated by neutralizing antibodies^5^. These immune-evading mutations often occur in the viral surface proteins, meaning that therapies targeting these proteins, such as vaccines or neutralizing antibodies, may lose effectiveness over time^7^. Viral variants may also result in the acquisition of resistance to drugs widely used for prophylaxis or treatment, e.g., the nearly universal amantadine resistance of recent epidemic and pandemic IAV strains^3^. Finally, the generation and selection of variants are favored in individuals who permit high and persistent levels of viral replication due to impaired adaptive immune responses. Therefore, there is a need for broad-spectrum antivirals (BSA) that target many strains or species, and that target viral replication at highly conserved areas that are under less selective pressure from adaptive immune responses. Such a BSA could provide an important line of defense for current and future pandemics.

The type VI-D CRISPR-Cas13d is an RNA-guided RNA ribonuclease that is directed by CRISPR-associated RNAs (crRNAs) to target and degrade specific RNA molecules^7,8^. It is a compact protein (967 amino acids) with high specificity and strong catalytic activity in human cells^7,8^. Cas13 viral-targeting RNases have potential advantages compared to traditional antivirals: targeting only relies on knowledge of the virus genome sequence; they can be developed quickly; they can be broadly effective if crRNAs are multiplexed; they can resist escaping mutations if targeting conserved sequence and/or to multiple loci^9–12^. As proof of concept, Cas13 variants have been used to target viral sequences of LCMV, RSV, IAV, VSV, PRRSV, HCV, and dengue virus in human cells^11,13–16^. We previously showed that Cas13d and virus-targeting crRNAs could prophylactically protect against IAV and SARS-CoV-2 sequences, a tool that we termed PAC-MAN (prophylactic antiviral CRISPR in human cells)^9,10^. Very recently, Cas13a has been tested to inhibit influenza virus or SARS-CoV-2 in animal models, and Cas13b has been shown to circumvent the mutational escape of SARS-CoV-2 through mismatch tolerance^11,12^. However, it remains unknown whether CRISPR can be used as a BSA to target many emerging variants of SARS-CoV-2 and other coronavirus strains. The ability to broadly protect against many respiratory virus strains simultaneously could be important not only for protection against currently circulating virus strains but also as part of a pandemic preparedness toolkit for future strains of concern.

Here, we demonstrate that Cas13d effectively targets and inhibits diverse strains of coronaviruses both as a prophylactic and a treatment, including SARS-CoV-2 and other coronaviruses. We present proof-of-principle work demonstrating how to optimize Cas13d and crRNAs for viral targeting, in addition to how it could be used as a BSA or in combination with other clinical small molecules to enhance protection from coronaviruses.

## Results

### Cas13d efficiently inhibits replication of diverse SARS-CoV-2 variants

**Fig. 1:**
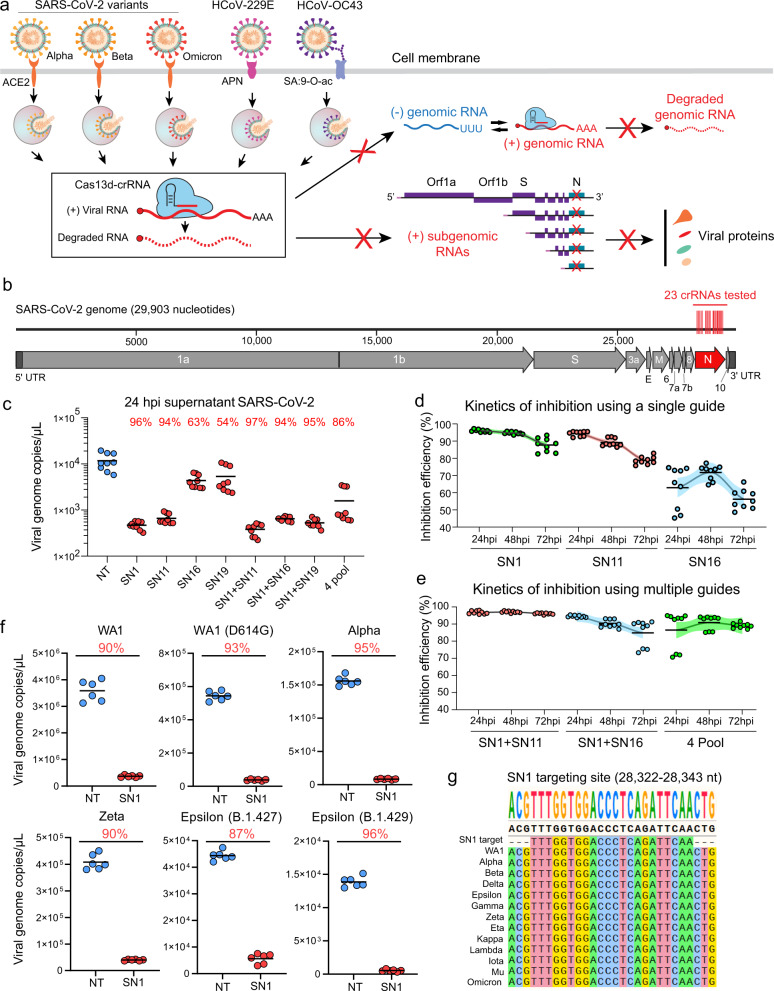
Cas13d broadly and effectively inhibits diverse SARS-CoV-2 variants. **a** Cartoon depicting the cell surface receptors involved in the viral entry for the coronaviruses SARS-CoV-2, 229E, and OC43, as well as potential viral replication steps that can be targeted by Cas13d. Cas13d is able to target both positive-sense genomic RNA as well as positive-sense subgenomic RNAs that are translated into proteins. The nested set of subgenomic RNAs is characteristic of the order *Nidovirales*, and means that the 3’-end of the genome that contains *N* is present in all subgenomic RNAs^19^. **b** SARS-CoV-2 genome structure and the location of 23 crRNAs that are targeting the *N* gene, and which were evaluated for antiviral activity. **c** Inhibition of SARS-CoV-2 by Cas13d. Vero E6 cells expressing Cas13d and a single crRNA or a pool of crRNAs, using a non-targeting (NT) crRNA as a control, were challenged with SARS-CoV-2 USA-WA1/2020. At 24 hpi, the virus amount in the supernatant was measured by reverse transcription quantification PCR (RT-qPCR); *n* = 3, *t* = 3. **d**, **e** A summary of the kinetics of single (**d**) or pooled (**e**) crRNAs and their effect on virus replication; *n* = 3, *t* = 3. Data presented as means ± SEM. **f** Inhibition of SARS-CoV-2 variants by Cas13d. Vero E6 cells expressing Cas13d were infected with SARS-CoV-2 variants at a multiplicity of infection (MOI) of 0.01. At 48 hpi, the virus titer in the supernatant was determined by RT-qPCR; *n* = 3, *t* = 2. **g** The genome sequences of some SARS-CoV-2 variants were aligned with the targeting sequence of crRNA SN1 using Mafft v7.480. *n* is the number of independent biological experiments. *t* is the number of technician replicates per biological replicate. All source data in this figure are provided as a Source data file. *P* values for the virus-targeting crRNAs are relative to NT (refer to Supplementary Data 3).

Coronaviruses are enveloped viruses with a large positive-sense RNA genome (~30 kb) that primarily infect the respiratory and gastrointestinal tract of animals^17^. Seven coronaviruses are known to infect humans, among which SARS-CoV, MERS-CoV, and SARS-CoV-2 have caused epidemics or pandemics in the past two decades, and HCoV-HKU1, HCoV-NL63, HCoV-229E, and HCoV-OC43 have caused seasonal common colds^18^. All coronaviruses studied to date have a similar life cycle^19^ (Fig. 1a). After the spike protein binds to the cell surface receptor (ACE2 for SARS-CoV-2, APN for HCoV-229E, or N-acetyl-9-O-acetylneuraminic acid receptor for HCoV-OC43) for cellular entry, the positive-sense genomic RNA is released into the cytosol and translated into protein machinery that is necessary for the generation of the negative-sense genomic and subgenomic RNAs. These negative-sense genomic RNAs are used as templates for the synthesis of new genomic RNAs, which in complex with the viral structural proteins produced by subgenomic RNAs, generate new virions to infect more cells^19^.

We previously designed crRNAs targeting SARS-CoV-2 RNA sequences and tested their efficiency for inhibition using a reporter system in human lung epithelial A549 cells^9,10^. Cas13d-induced target RNA cleavage can be easily re-directed to the viral RNAs of SARS-CoV-2 variants and other endemic human coronavirus strains, inhibiting the replication and transcription of viral RNAs and suppressing viral protein expression (Fig. 1a). To verify that *Ruminococcus flavefaciens XPD3002* Cas13d and the reporter-validated crRNAs were functional against SARS-CoV-2 virus, we screened 23 crRNAs targeting the nucleoprotein (*N* gene) for inhibiting viral replication by measuring viral genome copies in the supernatant of infected cells (Fig. 1b, c and Supplementary Fig. 1a, b). We chose *N* gene as a focus because it is one of the more conserved coronavirus genes. In addition, the nested structure of *Nidovirales* subgenomic RNAs means that the 3’ end of the genome is present in all subgenomic RNAs, which could potentially lead to a greater decrease in viral protein production after Cas13d cleavage of its target *N* gene sequence (Fig. 1a)^20^. We transduced African green monkey-derived kidney epithelial Vero E6 cells stably expressing NLS-Cas13d with non-targeting (NT) or virus-targeting crRNAs, and 2 days later challenged cells with the SARS-CoV-2 strain USA-WA1/2020 (hereafter referred to as WA1). The virus titer in the supernatant was determined by reverse transcription-quantitative PCR (RT-qPCR).

Compared with the NT crRNA, crRNAs targeting the *N* gene inhibited virus titer from 0 to 80% (Supplementary Fig. 1b). We validated the top-performing crRNAs individually or in combinations, and found that two crRNAs, SN1 and SN11, inhibited viral titer by 96% and 94% at 24 h post infection (hpi), respectively (Fig. 1c). The inhibition effect at 48 or 72 hpi was consistent with that seen at 24 hpi (Supplementary Fig. 1c). The combination of SN1 and SN11 slightly increased the inhibition efficiency to 97%, whereas a combination of four crRNAs (SN1 + SN11 + SN16 + SN19), exhibited a slightly decreased inhibition compared to SN1 and SN11 alone (Fig. 1c). We also quantified the viral RNA titer in cell lysates and found that most crRNAs except for SN16 showed a comparable or better inhibition of viral RNA than was observed in supernatants (Supplementary Fig. 1d). The cells with targeting crRNAs were visibly protected from SARS-CoV-2-induced cytopathic effects (CPE), as assessed by cell morphology and density (Supplementary Fig. 1e). The observed viral inhibition was highly dependent on the dosage of on-target crRNA lentivirus delivered to the Vero E6 cells, suggesting that cellular crRNA concentration is likely a limiting factor (Supplementary Fig. 1f). By measuring viral inhibition over time, we observed that while individual crRNAs showed reduced inhibition over time (Fig. 1d), combinations of crRNAs maintained their inhibition efficiency from 24 to 72 hpi (Fig. 1e).

Since the beginning of the pandemic, many new SARS-CoV-2 variants have evolved and become prominent forms of circulating virus^21^. Some of these have shown increased transmissibility, risk of death, and decreased sensitivity to convalescent sera and neutralizing antibodies^22–26^. We determined whether using the most potent crRNA (SN1) targeting the WA1 variant could inhibit different SARS-CoV-2 variants, including D614G, Alpha, Zeta, Epsilon (B.1.427), and Epsilon (B.1.429). We challenged Vero E6 cells with each of these variant strains and saw that Cas13d inhibited all variants by close to or more than 90% (Fig. 1f). We also aligned the genomic sequence of some representative SARS-CoV-2 variants (including Delta and Omicron) in the *N* gene region targeted by SN1, and found no mutations at the target site across any of the examined strains, indicating that these strains could potentially be inhibited by Cas13d with similar potency (Fig. 1g). We further retrieved sequences of 9,692,670 SARS-CoV-2 isolates from the Global Initiative on Sharing All Influenza Data (GISAID) database^27^ (as of 03/16/2022). SN1 is able to perfectly target 95.5% of SARS-CoV-2 isolates, regardless of the variations in different SARS-CoV-2 clades as identified in Nextstrain^28^. These were consistent with the fact that the *N* gene is more conserved than the spike gene^29^. This indicates that the conserved viral sequence targeted by Cas13d is not prone to mutations that enhance transmissibility or escape from immune recognition.

### Cas13d can potently inhibit other human coronavirus species

**Fig. 2:**
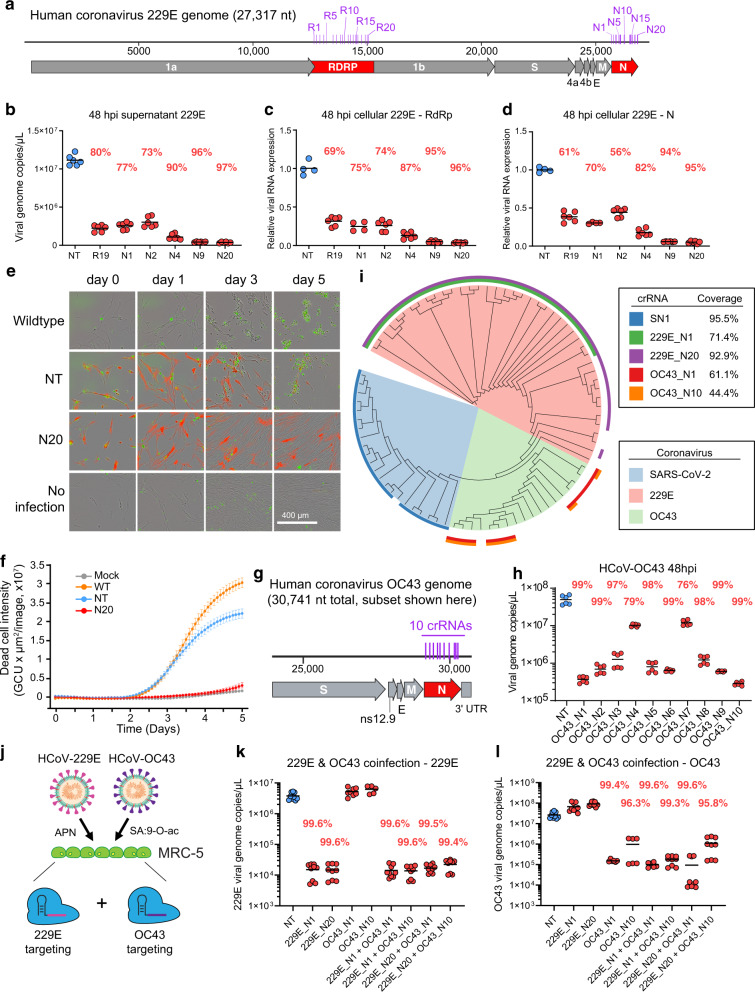
Cas13d strongly inhibits the human coronaviruses HCoV-229E and HCoV-OC43, singly and together. **a** The genome structure of 229E. Includes the locations of the 20 crRNAs targeting the *RdRp* gene and the 20 crRNAs targeting the *N* gene. **b**–**d** Inhibition of 229E by Cas13d. At 48 hpi, the virus titer in the supernatant (**b**) was measured by RT-qPCR. Viral RNA abundance in cell lysates was quantified using two sets of primers targeting *RdRp* (**c**) and *N* (**d**) gene, respectively; *n* = 3, *t* = 3. **e**, **f** Cell viability after 229E challenge was measured using the Incucyte live imaging system. **e** Incucyte images showing brightfield and fluorescence overlay for selected time points. Dead cells are in green and Cas13d+ cells are in red; *n* = 3. **f** Quantification of the signal from dead cells over time; *n* = 3 wells per group, 9 images per well, per time point. Data presented as means ± SEM. **g** The genome structure of OC43 and the locations of the 10 *N* gene-targeting crRNAs. **h** Inhibition of OC43 by Cas13d. At 48 hpi, the viral genome copies in the supernatant were measured by RT-qPCR; *n* = 2, *t* = 3. **i** Phylogenetic tree of representative strains of SARS-CoV-2, 229E, OC43. The outer ring shows coverage by each of the top crRNAs targeting these three coronaviruses. **j**–**l** Co-infection of 229E and OC43 in MRC-5 cells expressing different combinations of crRNAs. **j** Schematic depicting the strategy to protect against both 229E and OC43 simultaneously. At 48 hpi, the 229E (**k**) or OC43 (**l**) virus titer in the supernatant was measured by RT-qPCR; *n* = 3, *t* = 3. *n* is the number of independent biological experiments. *t* is the number of technician replicates per biological replicate. All source data in this figure are provided as a Source data file. *P* values for virus-targeting crRNAs (refer to Supplementary Data 3) are relative to NT. APN aminopeptidase N, SA:9-O-ac 9-O-acetyl-modified sialic acid.

We next tested whether Cas13d was able to inhibit other human endemic coronavirus strains. We targeted HCoV-229E (229E) and HCoV-OC43 (OC43), two common cold-causing coronaviruses. Using a similar strategy for screening crRNAs as above, we designed crRNAs targeting not only the *N* gene of 229E but also *RdRp*, which is another highly conserved gene (Fig. 2a). The crRNAs were screened by transducing human lung MRC-5 fibroblast cells with NLS-Cas13d and individual crRNAs and challenging with 229E or OC43 2 days later. For 229E, we performed microscopic imaging to qualitatively measure the CPE for each crRNA 3 days after challenge (Supplementary Fig. 2a). We then collected the supernatant from cells showing decreased CPE relative to the NT crRNA control and determined the virus titer by RT-qPCR (Supplementary Fig. 2b). Compared to the negative control, the best performing crRNAs strongly inhibited 229E replication by up to 95%.

We next validated top crRNAs of 229E and saw consistent results with the screen. The best crRNA, N20, inhibited 97% of viral genome production at 48 hpi (Fig. 2b). The viral RNA load in cell lysates was quantified using two sets of primers targeting *RdRp* and *N* genes, respectively, which detected both viral genomic RNA as well as viral subgenomic RNAs. We saw viral RNA reduction in the cell lysates consistent with the reduction of viral genome copy number in the supernatant (Fig. 2c, d). We also used the Incucyte live-cell imaging system to quantify cell survival after 229E infection in a 5-day time course. While almost all wild-type cells and cells expressing the NT crRNA died, the N20 crRNA sustained cell survival to a similar level as mock-infected cells across all 5 days (Fig. 2e, f and Supplementary Movies 1–4). Since *N*-targeting crRNAs were in general more effective for 229E, we designed 10 *N*-targeting crRNAs for inhibiting OC43 (Fig. 2g). We measured the OC43 virus titer in the supernatant by RT-qPCR at 48 hpi (Fig. 2h). The most effective crRNAs strongly inhibited OC43 replication by up to 99%.

We next explored the idea that Cas13d could provide BSA protection against human-targeting coronaviruses. We first performed a bioinformatic analysis to examine how well our best crRNAs were predicted to target multiple strains for each of the three coronavirus species we tested (Fig. 2i). We found that our top crRNAs, which all target the highly conserved *N* gene of their respective target species, were able to target 44–95% sequenced strains for each of the three coronavirus species we examined. We further tested whether Cas13d was able to inhibit co-infections of 229E and OC43 (Fig. 2j). While the crRNAs of each virus can only inhibit their corresponding virus, their combinations were able to inhibit both viruses at high efficacies of up to >99% (Fig. 2j–l). In summary, these data show that Cas13d is not only able to strongly protect against multiple coronaviruses but that it can also be multiplexed to protect against multiple viruses simultaneously.

### Characterization of the determinants of Cas13d antiviral performance in human cells

**Fig. 3:**
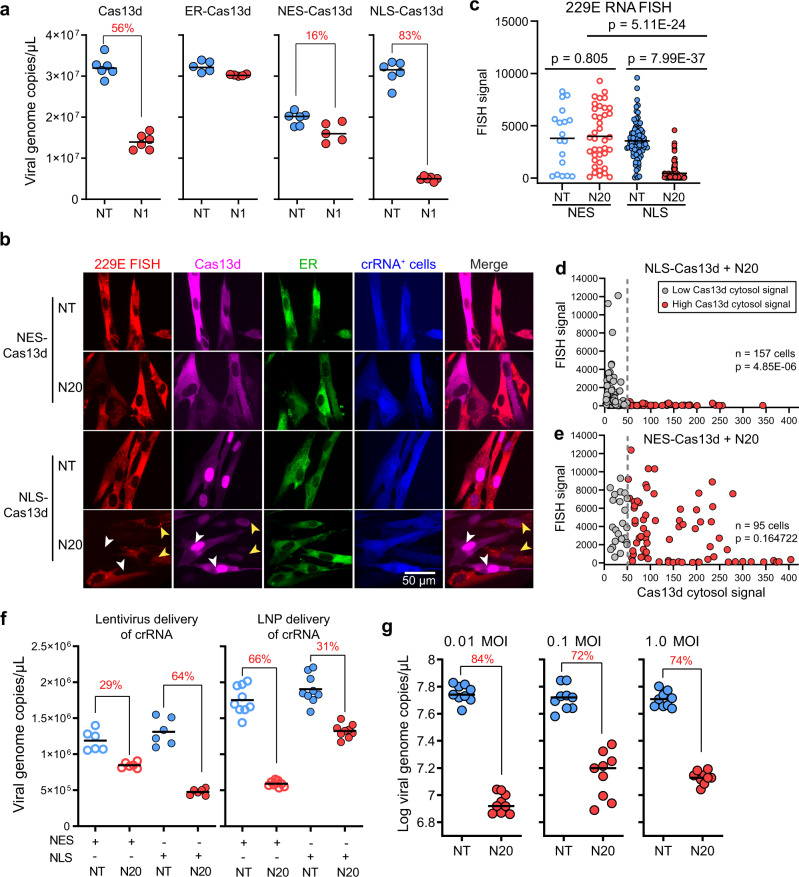
Characterization and optimization of Cas13d protection against coronavirus infection. **a** MRC-5 cells, transduced with the indicated Cas13d (with or without a subcellular location signal tag) and the NT or N1 crRNA, were challenged with 229E. Viral titer was determined by RT-qPCR; *n* = 3, *t* = 2. **b** Fluorescent microscopy of MRC-5 cells expressing mCherry-fused NES or NLS-Cas13d along with the NT or N20 crRNA, infected by 229E and fixed at 24 hpi. The 229E viral RNA was stained with RNA FISH probes labeled with AF647. **c** FISH signal (229E viral RNA abundance) mean intensity of each cell. All cells quantified were mCherry (Cas13d) and blue fluorescent protein (BFP; indicates crRNA^+^ cells) double positive. **d**, **e** The FISH signal and cytosol mCherry (Cas13d) signal measured from cells expressing NLS-Cas13d and N20 (**d**) and cells expressing NES-Cas13d and N20 (**e**). **f** MRC-5 cells, expressing NES or NLS-Cas13d, were delivered with indicated crRNA using lentivirus or lipid nanoparticle (LNP), and challenged with 229E. At 24 hpi, viral RNA in the supernatant was measured by RT-qPCR; *n* = 3 (for lentiviral delivered crRNA, *t* = 2; for LNP delivered crRNA, *t* = 3). **g** Cas13d antiviral activity against 229E infection at different MOIs; *n* = 3. *n* is the number of independent biological experiments. *t* is the number of technician repeats per biological replicate in the RT-qPCR assay. All source data in this figure are provided as a Source data file. *P* values are listed in supplementary Data 3, calculated by two-tailed Student’s *t* test.

We next characterized factors that determine Cas13d antiviral performance in cells. We previously used nuclear-localized NLS-Cas13d for targeting lentivirally encoded SARS-CoV-2 reporters, as it has been used for mRNA inhibition in mammalian cells^10^. However, since coronavirus RNA replication and transcription occur only in the cytoplasm and predominantly in the double-membrane vesicles (DMVs) derived from the endoplasmic reticulum (ER)^19^, we hypothesized that a Cas13d fused with a nuclear export sequence (NES-Cas13d) or an ER localization sequence (ER-Cas13d) might work more effectively for targeting coronavirus RNAs. Since coronaviruses have a conserved life cycle, we chose 229E as a model system to study how Cas13/crRNA delivery and localization characteristics affect viral inhibition^19^. We characterized 229E coronavirus inhibition in MRC-5 cells using Cas13d without a subcellular localization tag, or Cas13d fused with NLS, NES, or ER tags, which were delivered alongside the crRNAs using lentivirus (Fig. 3a). Surprisingly, NLS-Cas13d had the greatest antiviral activity, while the Cas13d without a tag, ER-Cas13d, and NES-Cas13d showed moderate or no viral inhibition effects.

To investigate why NLS-Cas13d performed better than NES-Cas13d, we used RNA fluorescent in situ hybridization (FISH) to evaluate the relationship between the abundance of viral RNA and the subcellular localization of Cas13d. We transduced the MRC-5 cells with mCherry-fused NLS-Cas13d or NES-Cas13d, along with an NT or N20 crRNA, which are encoded in a vector expressing blue fluorescent protein (BFP) (Supplementary Fig. 1a). The cells were challenged with 229E and fixed at 24 hpi. The 229E virus RNA was then stained with a set of FISH probes (Fig. 3b). The NES-Cas13d cells expressing the NT or N20 crRNAs showed no difference in 229E viral RNA abundance (Fig. 3b, c). In contrast, the N20 crRNA significantly inhibited viral RNA replication compared to the NT crRNA in the NLS-Cas13d cells (Fig. 3b, c). NLS-Cas13d/N20 cells showed statistically lower viral RNA abundance than the NES-Cas13d/N20 cells (Fig. 3c). Importantly, we observed that NLS-Cas13d was leaky and localized in both the nucleus and the cytoplasm in some cells (Fig. 3b, white arrows and Supplementary Fig. 3c, d). Such cells showed a much lower 229E signal than those that only had nuclear-localized Cas13d (compare white to yellow arrows, Fig. 3b). Statistically, we observed a clear inverse correlation between the 229E FISH signal and cytoplasmically localized mCherry-Cas13d signal for NLS-Cas13d expressing cells, but not for NES-Cas13d cells (Fig. 3d, e).

To account for these observations, we hypothesized that lentivirally expressed crRNAs were not efficiently exported to the cytoplasm and that only the NLS-Cas13d was able to complex with crRNA molecules due to its co-localization with crRNAs in the nucleus. To test this, we designed an experiment where MRC-5 cells were transduced with either NES-Cas13d or NLS-Cas13d, and delivered crRNAs either through lentivirus transduction or lipid nanoparticle (LNP) transfection of synthesized crRNAs. We hypothesized that lentivirus would integrate into the genome and express the crRNA in the nucleus, potentially with a low efficiency of cytoplasmic export, while LNP transfection would deliver the crRNA mostly to the cytoplasm (Supplementary Fig. 3c, d). Consistent with our hypothesis, NLS-Cas13d showed better viral inhibition than NES-Cas13d with lentiviral delivery of crRNAs (Fig. 3f). In contrast, NES-Cas13d exhibited better viral inhibition than NLS-Cas13d when using LNP to deliver crRNAs (Fig. 3f). This data suggests that Cas13d activity is highly dependent on the colocalization of Cas13d protein and the crRNA, an effect that should be considered when choosing delivery methods (Supplementary Fig. 3e).

We next characterized how antiviral efficacy was dependent on viral titer. We challenged MRC-5 cells expressing NLS-Cas13d with different multiplicities of infection (MOI) of 229E. We found that Cas13d inhibited virus replication and protected cells from death across two orders of magnitude of MOIs ranging from 0.01 to 1.0 (Fig. 3g and Supplementary Fig. 3a). While Cas13d was more effective at a lower MOI (84% inhibition), it remained effective at non-physiological MOIs of 0.1 and 1.0 (72% and 74% inhibition, respectively). As with our SARS-CoV-2 results (Supplementary Fig. 1e), 229E viral inhibition was dependent on the dosage of Cas13d and the crRNA (Supplementary Fig. 3b). Together, our data highlight the importance of Cas13d/crRNA co-localization in the cytoplasm as well as the high expression level of both Cas13d and crRNA for achieving high antiviral activity.

### crRNAs targeting viral sequences are highly specific

We next explored whether the targeting crRNAs affected the host cell gene expression. To do this, we used a type of LNP, termed a lipitoid, to transiently transfect human A549 lung carcinoma cells with the Cas13d mRNA and in vitro synthesized crRNA(s) (Supplementary Fig. 7a). We chose a lipid nanoparticle system to study off-target effects since this represents the strategy that will most likely be developed for clinical administration. The lipitoid agent contains a peptoid backbone and a hydrophobic lipid moiety that facilitates cell uptake^30^. At 48 hpi, we isolated cellular RNA and performed whole-cell RNA sequencing (RNA-seq). We observed high reproducibility between biological replicates (Supplementary Fig. 4a). We examined the off-target effects of viral-targeting crRNA for SARS-CoV-2 or 229E as well as the NT crRNA by comparing their transcriptome profiles to that of cells expressing Cas13d alone, and we saw highly similar gene expression profiles (*R*^2^ = 0.978, 0.977, 0.979, 0.979, 0.979, 0.977, respectively; Supplementary Fig. 4b, c). Furthermore, co-delivery of Cas13d and two targeting crRNAs showed a very similar transcriptome profile to that of the Cas13d alone (*R*^2^ = 0.977; Supplementary Fig. 4c). These data suggest that Cas13d plus antiviral crRNAs are specific, with a minimal impact on host cell transcriptomes.

### Cas13d interacts with antiviral small-molecule drugs to enhance coronavirus inhibition

**Fig. 4:**
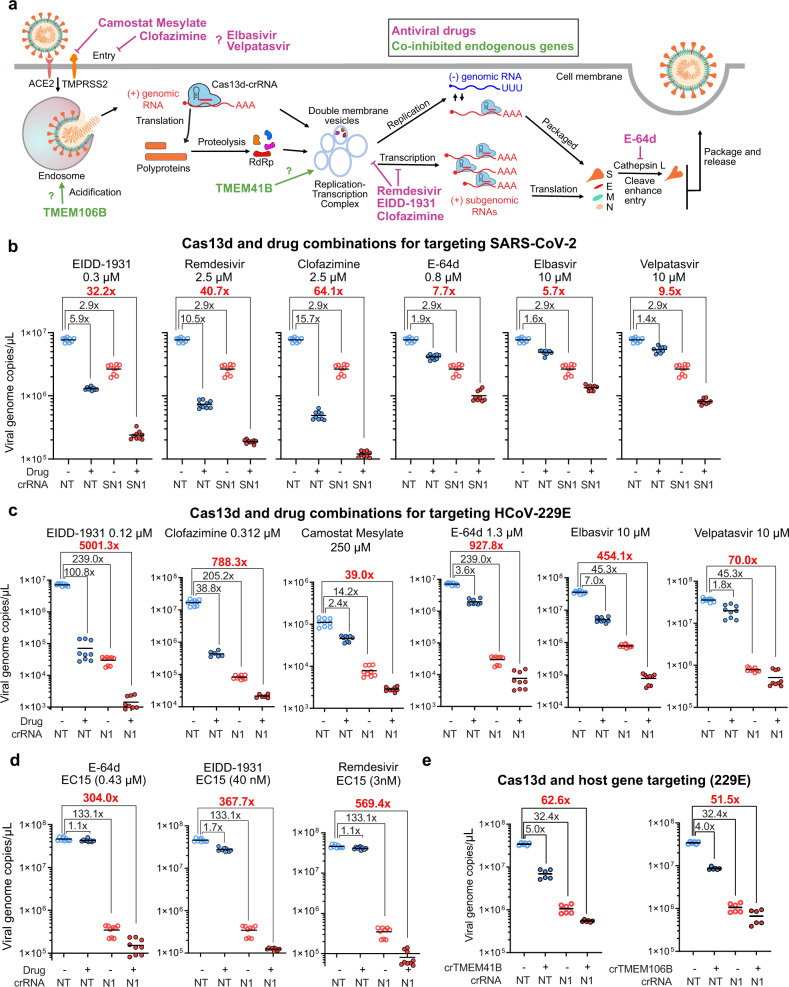
Combining viral-targeting Cas13d with small-molecule drugs or host-targeting crRNAs results in enhanced inhibition of coronaviruses. **a** The life cycle of coronavirus and known/hypothesized mechanism of action of antivirals targeting the different pathways involved in viral replication. **b** Combinations of Cas13d with antiviral small-molecule compounds and their effect on inhibition of SARS-CoV-2. Vero E6 cells expressing Cas13d and NT or SN1 crRNA were pre- and post-treated with indicated drug and challenged with USA-WA1/2020 SARS-CoV-2 at an MOI of 0.01. At 48 hpi, the viral genome copies in the supernatant were determined by RT-qPCR; *n* = 3, *t* = 3. **c** Combinations of Cas13d with small-molecule antivirals on inhibition of 229E virus. MRC-5 cells expressing Cas13d and NT or N1 crRNA were pre- and post-treated with the indicated antiviral drugs at the indicated dose and infected with 229E at an MOI of 0.01. The virus titer was measured at 48 hpi by RT-qPCR; *n* = 3, *t* = 3. **d** The combination of Cas13d with several drugs at a dose of EC15 on inhibition of 229E virus; *n* = 3, *t* = 3. **e** Combination of 229E- and host gene-targeting crRNAs on the inhibition of 229E; *n* = 3, *t* = 2. *n* is the number of independent biological experiments. *t* is the number of technician replicates per biological replicate in the RT-qPCR assay. All source data in this figure are provided as a Source data file. *P* values are listed in supplementary Data 3.

We next sought to enhance the antiviral activity of Cas13d by combining it with small-molecule drugs that are in development or in clinical use for treating coronavirus infection (Fig. 4a). To do this, we chose drugs that target various pathways in the coronavirus life cycle, including drugs targeting viral entry (camostat mesylate, E-64d, and clofazimine), viral RNA synthesis (EIDD-1931, remdesivir, and clofazimine), and drugs showing synergy with remdesivir (elbasivir and velpatasvir)^31–36^. We first characterized the cytotoxicity of the selected antiviral drugs with serial dilutions and determined the concentration for half-maximal response (EC50) of each drug for inhibiting 229E infection in MRC-5 cells (Supplementary Fig. 5a).

We next tested how combinations of Cas13d/crRNA with small-molecule drugs affected coronavirus infection. To achieve a better inhibition dynamic range, we decreased the dose of lentivirus of Cas13d and crRNA in the combination tests. All the tested drugs, including EIDD-1931, remdesivir, clofazimine, E-64d, elbasvir, and velpatasvir, showed greatly enhanced SARS-CoV-2 inhibition when combined with Cas13d and an on-target crRNA (Fig. 4b). For example, we saw enhanced antiviral activity when using EIDD-1931 and Cas13d/SN1 together. While EIDD-1931 or Cas13d/SN1 alone reduced virus titer by 5.9-fold and 2.9-fold, respectively, using both reduced the viral titer by 32.2-fold (Fig. 4b). The enhanced antiviral activity was also observed for targeting 229E using Cas13d and antiviral small-molecule drugs (Fig. 4c). Notably, using Cas13d/crN1 and EIDD-1931 together reduced the 229E viral titer by more than 5000-fold, whereas Cas13d/crN1 or EIDD-1931 alone respectively showed 238.9-fold or 100.8-fold of inhibition. The same effect was also observed using another crRNA (N20) and the indicated small-molecule drugs (Supplementary Fig. 5b). We expect that the greatly enhanced antiviral effect of Cas13d and small-molecule antiviral drugs will be a useful strategy to more effectively suppress viral replication in the future.

One limitation of small-molecule antiviral drugs is their cytotoxicity at a high dosage. We envision the combinatorial effects between Cas13d/crRNA and drugs can effectively reduce the drug dosage and yet still achieve a high antiviral effect. Interestingly, while E-64d, EIDD-1931, and remdesivir showed almost no antiviral activity at a low dosage (EC15), combinations with Cas13d/on-target crRNA showed a synergistic inhibition of viral replication, which was greater than the individual effects of drug or Cas13d/on-target crRNA multiplied together (Fig. 4d and Supplementary Fig. 5c).

### Simultaneous Cas13d-mediated inhibition of coronavirus RNA and host genes enhances antiviral activity

In addition to being used as an antiviral, Cas13d can be used to repress the expression of endogenous genes, many of which have no available small-molecule inhibitors or antibodies. *TMEM41B* and *TMEM106B* are pro-viral host factors for coronavirus replication that were recently identified as part of a genome-wide CRISPR-Cas9 knockout screen^37^. Using Cas13d and crRNAs simultaneously targeting the viral RNA and the mRNA of *TMEM41B* or *TMEM106B* greatly enhanced 229E inhibition (Fig. 4e), suggesting Cas13d can be used as an “all-in-one” system to target either viral and/or host genes as a versatile antiviral strategy.

### Cas13d treatment inhibits established viral infection, alone or in combination with small molecules

**Fig. 5:**
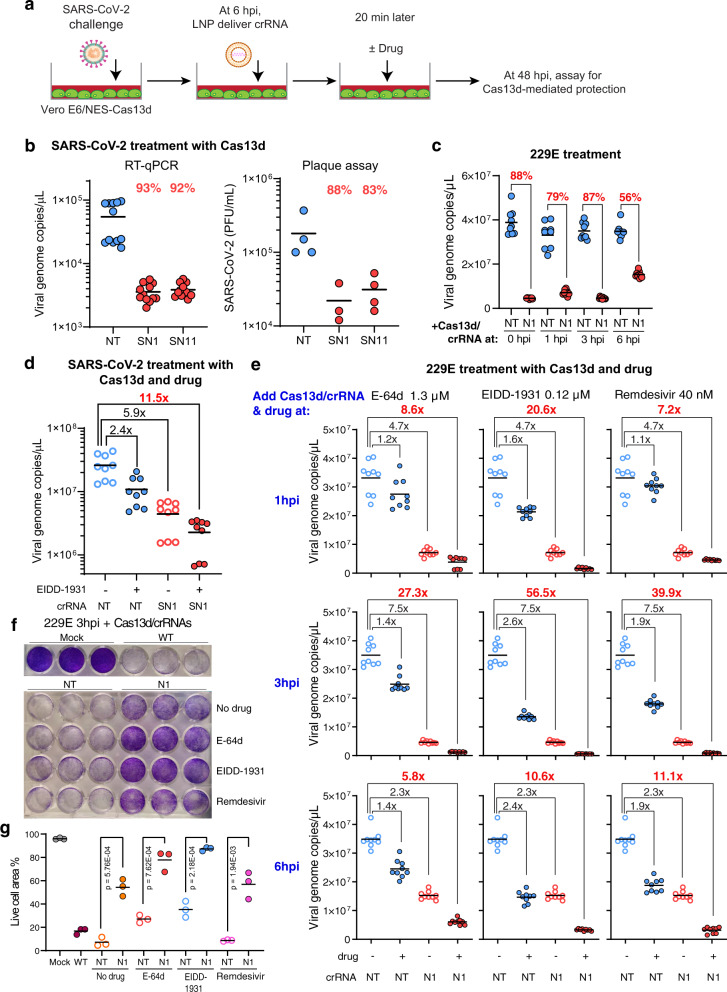
Cas13d antivirals can treat established viral infection in cell lines. **a** Schematic of the antiviral treatment of SARS-CoV-2 infection in Vero E6/NES-Cas13d cells. **b** Treatment test of Cas13d targeting SARS-CoV-2 in Vero E6/NES-Cas13d cells. At 6 hpi, the cells were transfected with crRNA (Synthego), NT or SN1, using LNP. At 48 hpi, the virus titer in the media was quantified using both RT-qPCR and plaque assays; *n* = 4, *t* = 3. **d** The antiviral drug EIDD-1931 was added to the media in combination with the Cas13d; *n* = 3, *t* = 3. **c**, **e**–**g** Treatment test of Cas13d targeting 229E in MRC-5 cells. At 0, 1, 3, and 6 hpi, the cells were transfected with NES-Cas13d mRNA (Trilink) and crRNA (Synthego) using lipofectamine MessagerMAX (Invitrogen). The indicated small-molecule antiviral drug was added to the media 20 min after the transfection. At 48 hpi, virus titer in the supernatant was measured by RT-qPCR. The viral genomic copies of Cas13d treatment with no drug (**c**) or combined with a small-molecule drug (**e**) are plotted separately; *n* = 3, *t* = 3. At 72 hpi, the cells were stained with crystal violet (**f**) and the percent vial cells were quantified using Fiji ImageJ (**g**); *n* = 3. *n* is the number of independent biological experiments. *t* is the number of technician replicates per biological replicate in the RT-qPCR assay. All source data in this figure are provided as a Source data file. *P* values are listed in supplementary Data 3, calculated by two-tailed Student’s *t* test.

We next sought to test whether Cas13d could be used as a treatment for established viral infection. To do this, we constructed a Vero E6 cell line which stably expresses NES-Cas13d (designated as Vero E6/NES-Cas13d). We infected Vero E6/NES-Cas13d cells with SARS-CoV-2 or infected MRC-5 cells with 229E, both at an MOI of 0.01 (Fig. 5a and Supplementary Fig. 6a). At 6 hpi, the Vero E6 cells were transfected with Synthego synthesized crRNAs NT, SN1, or SN11. The virus titer was decreased by >90% by RT-qPCR and by close to 90% by plaque assay at 48 hpi, indicating Cas13d is effective in suppressing SARS-CoV-2 replication (Fig. 5b and Supplementary Fig. 6b). At 0, 1, 3, or 6 hpi, MRC-5 cells were transfected with NES-Cas13d mRNA and crRNAs NT or N1. When Cas13d/crN1 was delivered at 0, 1, 3, or 6 hpi, the virus titer was decreased by 88%, 79%, 87%, and 56%, respectively, relative to the NT crRNA, suggesting it was effective in controlling early infection with coronaviruses (Fig. 5c). When Cas13d/crRNA treatment was combined with a small-molecule antiviral drug (either E-64d, EIDD-1931, or remdesivir), the inhibitory effect of Cas13d was greatly increased (Fig. 5d–g and Supplementary Fig. 6a, b). For example, combinations of Cas13d with EIDD-1931 increased the treatment inhibitory effect on SARS-CoV-2 to 11.5-fold, while drug alone was 2.4-fold and Cas13d/SN1 alone was 5.9-fold (Fig. 5d). It increased the treatment inhibitory effect on 229E to 56.5-fold when the cells were treated at 3 hpi, while drug alone was 2.58-fold and Cas13d/N1 alone was 7.5-fold (Fig. 5e). These results suggest a strong synergistic effect using Cas13d/crRNA and drugs for treatment. With treatment at 3 hpi, while most wild-type cells or cells transfected with Cas13d/NT crRNA were killed by 229E infection, 54% of cells were alive using Cas13d/N1 crRNA alone and 78% or 87% of cells alive when combining Cas13d/N1 crRNA with E-64d or EIDD-1931, respectively (Fig. 5f, g). Increased cell survival was also observed when the cells were treated at 1 and 6 hpi (Supplementary Fig. 6c, d).

### Characterization of different delivery methods for Cas13d and crRNAs

To implement a therapeutic delivery system for Cas13d/crRNAs, we explored mRNA delivery via LNPs. Previously studies have shown that lipitoids are effective for mRNA delivery in primary cell types due to their resistance to proteolytic degradation and diverse side-chain chemistry^38–40^. We explored the delivery of Cas13d mRNA and synthesized crRNAs using the lipitoid delivery agent DMPE ((NaeNmpeNmpe)_3_; Supplementary Fig. 7a).

We characterized lipitoid-mediated Cas13d mRNA delivery in human lung A549 cells. We added lipitoid-packaged mCherry-NES-Cas13d mRNA to A549 cells, incubated for 45 min, and washed cells. One day after transfection, we observed more than 62% mCherry^+^ cells (Supplementary Fig. 7b). We also tested lipitoid-packaged crRNA transfection. Interestingly, crRNA transfection reached 100%, likely due to its small size. In MRC-5 cells, the transfection of mCherry-Cas13d mRNA with lipitoid was similar to the delivery in A549 cells (Supplementary Fig. 7c). We also used nebulization to deliver lipitoid-packaged Cas13d mRNA. Interestingly, nebulization enhanced the delivery efficiency of lipitoid-packaged mCherry-Cas13d from 59.1% to 66.5% and increased the delivery of mCherry mRNA from 93.1 to 99.8% (Supplementary Fig. 7d, e). We further characterized the long-term storage of lipitoid-packaged Cas13d for transfection. Among all conditions tested, storage at −80 °C combined with 25% glycerol preserved the transfection efficiency of the lipitoid delivery agent without loss of activity (Supplementary Fig. 7f, g). In both A549 and MRC-5 cells, we found a gradual decline of mCherry-Cas13d expression over time (Supplementary Fig. 7h). Overall, we were able to observe that most cells continued to express mCherry-Cas13d even 6 days after transfection.

We also characterized the use of influenza A virus (IAV)-pseudotyped lentivirus for Cas13d delivery to lung cells. Previous studies have shown that lentivirus pseudotyped with hemagglutinin and neuraminidase from IAV can efficiently deliver transgenes to the lungs of mice^41–45^. We sought to leverage this technology to see if we could efficiently deliver Cas13d antivirals to cells for protection against 229E. Of note, typical VSV-G pseudotyped lentivirus utilizes the low-density lipoprotein receptor (LDLR) to enter cells^46^; however, this receptor is typically located on the basolateral membrane of airway cells and is thus inaccessible to lentivirus without harsh pre-treatment^45,47,48^. Pseudotyping lentivirus with IAV hemagglutinin allows lentivirus to use sialylated glycoproteins on the apical membrane to enter cells and deliver its genetic payload. We found that IAV-pseudotyped lentivirus can efficiently deliver Cas13d antivirals to human primary bronchial epithelial cells (hPBEC) and MRC-5 cells in vitro (Supplementary Figs. 7i, 8d). We also observed similar strong protection against 229E when Cas13d and crRNAs were delivered with IAV-pseudotyped lentivirus (Supplementary Fig. 7j). This suggests another promising modality for delivering Cas13d antivirals to human lung cells.

### Cas13d/crRNA is an effective coronavirus treatment in primary human respiratory cultures

To test the ability of Cas13d to act as an antiviral in primary lung cells, we tested Cas13d against virus replication in air–liquid interface (ALI) cultures derived from human primary bronchial epithelial cells (hPBECs). ALI cultures are an in vitro model of the human airway that can be expanded and differentiated into a pseudostratified epithelium that recapitulates the architecture and many of the cell types of the human lung^49^. We first established that 229E and SARS-CoV-2 can successfully replicate in ALI cultures (Supplementary Fig. 8a, b). We also verified that the lipitoid delivery agent was able to deliver crRNAs to ALI cultures at nearly 100% efficiency (Supplementary Fig. 8c).

**Fig. 6:**
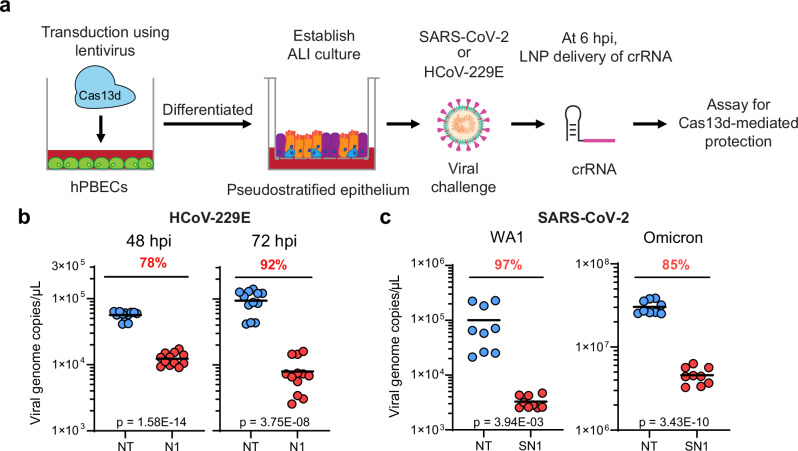
Cas13d antivirals can treat established viral infection in human primary lung epithelial cultures. **a** Scheme used for antiviral treatment of air–liquid-interface (ALI) cultures of human primary bronchial epithelial cells (hPBECs). hPBECs were transduced with NES-Cas13d and differentiated to ALI cultures. The ALI cultures were infected with 229E at an MOI of 0.05, with SARS-CoV-2 WA1 at an MOI of 0.6, or with Omicron at an MOI of 0.1. At 6 hpi, the culture was transfected with crRNA using LNP. At 48, 72 hpi, the apical surfaces of ALI cultures were washed with PBS and the wash solution was collected for measuring virus titer. **b** The titer of 229E virus released on the apical surface of ALI cultures was determined by RT-qPCR; *n* = 4, *t* = 3. **c** The titer of SARS-CoV-2 virus, including WA1 and Omicron strains, was determined at 48 hpi; *n* = 3, *t* = 3. *n* is the number of independent biological experiments. *t* is the number of technician replicates per biological replicate in the RT-qPCR assay. All source data in this figure are provided as a Source data file. *P* values are listed in supplementary Data 3, calculated by two-tailed Student’s *t* test.

To test Cas13d function in ALI cultures, we transduced hPBECs with VSV- or IAV-pseudotyped lentivirus that encoded NES-Cas13d (Supplementary Fig. 8d, e), differentiated these hPBECs into pseudostratified epithelial ALI cultures, and then challenged these cultures with 229E at an MOI of 0.05, with SARS-CoV-2 WA1 at an MOI of 0.6, or with SARS-CoV-2 Omicron variant at an MOI of 0.1 (Fig. 6a). At 6 hpi, the cells were transfected with crRNAs using LNPs. The titer of the released virus was determined by washing the apical surfaces of ALI cultures with PBS and performing RT-qPCR. We observed that the virus titer of 229E was reduced by 78% at 48 hpi and by 92% at 72 hpi and the virus titer of SARS-CoV-2 WA1 was reduced by 54% at 24 hpi and 97% at 48 hpi (Fig. 6b, c and Supplementary Fig. 8f). Excitingly, the crRNA SN1, which was designed before the emergence of Omicron, can effectively reduce the Omicron variant copy number by 70% at 24 hpi and 85% at 48 hpi, respectively (Fig. 6c and Supplementary Fig. 8f). These results indicate a strong treatment effect from Cas13d/crRNA and that a crRNA targeting an evolutionarily conserved sequence maintains a consistent inhibitory effect on emerging variants.

## Discussion

Here we provide substantial evidence that CRISPR-Cas13d can work efficiently as a BSA agent against diverse SARS-CoV-2 variants, including Omicron, as well as endemic human respiratory coronavirus strains. We demonstrated that Cas13d and selected top-performing crRNAs can robustly inhibit diverse coronaviruses. We characterized the determinants of Cas13d-mediated antiviral efficiency and found that subcellular colocalization of Cas13d protein and crRNAs is critical for efficient viral targeting. We found that viral-targeting crRNAs have no detectable off-target changes on the host transcriptome. Notably, Cas13d was able to provide protection against co-infection by multiple human coronaviruses. In addition, Cas13d greatly enhanced the antiviral activity of diverse small-molecule drugs that act by distinct mechanisms, and the best Cas13d-drug combination resulted in a >5000-fold inhibition of viral titer. Using LNP-mediated RNA delivery, Cas13d-crRNA was effective as a treatment post infection for coronavirus-infected human cell lines and primary lung epithelium. We conclude that Cas13d offers a programmable and effective antiviral strategy for broadly protective coronavirus prophylaxis and treatment.

Our study establishes several key advantages of CRISPR antivirals. Since the outbreak of the COVID-19 pandemic, new SARS-CoV-2 variants, including the highly infectious Omicron variant, have emerged with significant mutations in the spike surface protein that greatly enhance infectivity and transmissibility^25,26,50–58^. This poses a major global healthcare and economic challenge, as viral variants of concern may emerge with dangerous resistance to the immunity generated by the current vaccines^59^. Some monoclonal antibodies have shown impaired binding to the spike protein of variants^51,54,60,61^. In contrast, the CRISPR-Cas13 approach targets genomic regions that are more conserved and thus more resistant to virus escaping mutations, as seen in our study. This Cas13d-based BSA concept offers a complementary strategy to existing vaccines that generally display a narrower range of variant protection.

Furthermore, since the genomic region targeted by our designed crRNAs is highly conserved across coronavirus strains, our broad-spectrum antiviral strategy can target current and potentially future pandemic strains. Indeed, using a pre-validated crRNA targeting WA1 we showed efficient inhibition of Omicron in human primary bronchial epithelial culture, highlighting the broad-spectrum nature of our approach. In addition to SARS-CoV-2, our strategy exhibited similarly high efficiency for inhibiting human endemic 229E and OC43 coronaviruses. Beyond these tested strains, many coronaviruses originating from animal species may become infectious in humans, as demonstrated by outbreaks of three coronavirus epidemics or pandemics in the past 20 years. There is a great need for new tools in our pandemic preparedness toolkit that can target highly conserved components of the viral life cycle that are orthogonal to vaccines, monoclonal antibodies, or antiviral drugs and that can buffer against viral mutations to escape immune responses.

Using our approach, multiple crRNAs targeting SARS-CoV-2 could be present in a crRNA cocktail to help prevent mutational escape. In addition, crRNAs could be included that are predicted to broadly target other pandemic-potential strains of coronaviruses. Interestingly, our CRISPR-Cas13 strategy can robustly inhibit simultaneous co-infection of two coronaviruses when two virus-targeting crRNAs are delivered to cells. This provides a model for a possible BSA that simultaneously protects against multiple respiratory RNA viruses. This would allow for an off-the-shelf antiviral treatment that could not only protect against multiple strains of SARS-CoV-2 but also “common cold” strains of coronavirus and/or IAV. Thus, our CRISPR-Cas13 strategy can help fill a need for therapeutic interventions that can be broadly protective and rapidly deployed against novel viral threats.

We also show in this study that Cas13d can markedly enhance prophylaxis and treatment effects when combined with small-molecule antiviral drugs. For example, a combination of Cas13d and EIDD-1931 decreased the 229E virus titer by almost 4 logs. Quantitatively, we observed either additive or synergistic effects between Cas13d and different drugs for inhibiting SARS-CoV-2 or 229E. Their combinations helped reduce the drug dose to achieve a strong effect. This provides a potential strategy to reduce drug-induced cytotoxicity, a common issue faced in the development of safe antiviral drugs.

Our experiments also revealed the importance of colocalizing Cas13d and crRNAs for more effective antiviral targeting in cells. We saw that different delivery methods may have distinct properties for crRNA localization. For example, while lentivirally transduced crRNAs are likely predominantly localized in the nucleus and may not be efficiently exported to the cytoplasm, lipid nanoparticle delivery localizes most crRNAs in the cytoplasm. Since coronavirus RNAs are localized in the cytoplasm, consideration of these factors is needed for optimal performance of Cas13-based antiviral therapies. We also provide RNA-seq data to show that Cas13d/crRNAs do not affect the human transcriptome when introduced into cells without coronavirus targets. If further translated therapeutically, it will be important to observe whether Cas13d exhibits collateral cleavage off-target effects after on-target viral cleavage.

While we have shown that Cas13d and on-target crRNAs can function well as an antiviral in cultured cell lines and human primary bronchial epithelial cultures, there is still a long path before this can be clinically translated, as well as some potential pitfalls to overcome. In the future, optimizing Cas13d/crRNA delivery using clinically relevant delivery formulations both in vivo and in mucous-producing human primary lung cell cultures such as the ALI system will be key. In addition, it will be important to determine appropriate dosage and treatment regimens; one potential issue is that repeated doses may result in immunogenicity against Cas13d and/or the delivery reagent, which will need to be accounted for. Some Cas13 proteins may also have collateral effects after on-target RNA cleavage, which should be examined or minimized before translating to the clinic. As Cas13 biology is rapidly advancing, compact, more effective, less immunogenic, and more specific RNA-targeting Cas proteins may be discovered in the future^62–64^, which would be better suited for clinical uses. If that is the case, we believe that the approach and pipeline for crRNA discovery and testing that we have optimized here for creating a BSA would be readily transferred to a new type of Cas molecule. Since our strategy has a high potential for therapeutic development, the safety and efficacy of CRISPR-based-antivirals and their putative delivery mechanisms need further preclinical tests in rodents, ferrets, and non-human primates prior to embarking on phase I clinical tests in humans. Although there are still several obstacles to overcome before CRISPR-based antivirals could be employed in clinical trials, it has substantial advantages as an antiviral therapy for broadly responding to emerging viruses.

## Methods

### Cell cultures, primary cells, viral strains

Human embryonic kidney (HEK293T, Cat# CRL-3216), A549 (Cat# CCL-185), MRC-5 (Cat# CCL-171), and Vero E6 (Cat# CRL-1586) cells were purchased from ATCC and cultured in 10% fetal bovine serum (Thermo Fisher Scientific) in DMEM (Thermo Fisher Scientific). Human primary bronchial epithelial cells (hPBEC) were purchased from ATCC (PCS300010) and maintained in PneumaCult-Ex Plus Medium (StemCell, Canada) supplemented with hydrocortisone (0.5 μg/ml). All cell lines were incubated at 37 °C in a 5% CO_2_ atmosphere.

Human coronavirus 229E (Cat# VR-740, ATCC) and OC43 (Cat# VR-1558, ATCC) were amplified once by inoculating a T75 flask of fully confluent MRC-5 cells and collecting cell culture supernatant at 48 h post infection (hpi) followed by centrifugation at 1500×*g* for 10 min to remove cell debris. The virus titer was determined using TCID_50_ assay. The virus was aliquoted, and stored at −80 °C for later use. All experiments involving the human coronavirus 229E were performed in a Biosafety Level (BSL) 2 laboratory.

Severe Acute Respiratory Syndrome-Related Coronavirus 2 (SARS-CoV-2), Isolate USA-WA1/2020, NR-52281, and Isolate hCoV-19/USA/MD-HP20874/2021 (Lineage B.1.1.529; Omicron Variant), NR-56461, contributed by Dr. Andrew Pekosz, were obtained from BEI Resources, NIAID, NIH. The SARS-CoV-2 variants D614G, Alpha, Zeta, Epsilon (B.1.427), and Epsilon (B.1.429) were isolated from patient swabs as described previously^65^. Briefly, the initial isolate was passed through a 0.45-µm syringe filter, then passaged twice in Vero E6 cells. For each passage, ~80% confluent T-175 flasks (Nunc, Roskilde, Denmark) of Vero E6 cells were inoculated with 5 μl of virus. Cytopathic effect (CPE) was monitored daily and flasks were frozen down when monolayers exhibited ~80% CPE. Thawed lysates were collected and cell debris was pelleted at 1500×*g* for 20 min. The clarified supernatant was aliquoted and the infectious virus was quantified by TCID_50_ assay, ranging from 1 × 10^6^ to 5 × 10^6^ TCID50 units/mL. Passage 2 virus was used for subsequent experiments. All experiments involving SARS-CoV-2 infection were done in the BSL3 facility at the University of California, Berkeley, and the BSL3 facility at Stanford University.

### crRNA design, cloning, and synthesis

The crRNAs were designed using the Cas13design tool^66^. The crRNA plasmids were cloned using standard restriction-ligation cloning. Forward and reverse oligos for each spacer (IDT) were annealed and inserted into a pHR backbone using T4 DNA Ligase (NEB). The backbone was modified from Addgene (#121514). Spacer sequences and predicted efficiency score for all crRNAs can be found in Supplementary Data 1. Some crRNAs were synthesized by Synthego using solid-phase phosphoramidite chemistry to contain 3’ Um modifications with additional 5’ Cy5 labeling^67^. A protected 3’-thiol moiety was installed using Thiol-Modifier C6 S-S (Cat# 10-1936-90E, Glen Research). Following cleavage, deprotection and isolation, the free thiol was liberated using dithiothreitol and then reacted with Cyanine5 Maleimide (Cat# 43080, Lumiprobe) following the manufacturer’s guidelines. Purity and identity were confirmed via HPLC/ESI-MS. These are listed in Supplementary Data 1. They were used in the treatment tests as described below.

### Lentivirus package and stable cell-line generation

To produce lentivirus, HEK293T cells were transiently transfected with the lentiviral plasmid, and packaging plasmids pCMV-dR8.91 and pMD2.G. After 72 h of transfection, the supernatant was passed through 0.45-μm filters, concentrated using Lenti-X concentrator (Cat# VC100, ALSTEM) at 4 °C overnight, and centrifuged at 1500×*g* for 30 min at 4 °C to collect virus pellets. The virus pellets were resuspended in cold culture medium for storage at −80 °C or transduction of cells. One well of a six-well plate (Corning) of Vero E6 cells at 50% confluency was transduced with the precipitated lentivirus encoding NLS- or NES-Cas13d. After 2–3 days of growth, the cell supernatant containing the virus was removed and the cells were expanded. Cells were then sorted for mCherry^+^ cells using a Sony SH800S cell sorter. The cells were expanded from single colonies.

### Quantitative reverse transcription PCR (RT-qPCR)

To quantify viral RNA abundance in the supernatant samples, RT-qPCR was performed with RNA standards synthesized using a TranscriptAid T7 High Yield Transcription Kit (Thermo Scientific Cat# K0441) from the DNA templates generated by RT-PCR from the SARS-CoV-2 and 229E viral RNA. For each sample, total RNA was isolated using a Quick-RNA Viral kit (Zymo Research Cat# R1035). RT-qPCR was performed using the GoTaq Probe 1-Step RT-qPCR System (Promega Cat# A6120). RT-qPCR primers and probes are listed in Supplementary Data 2.

To quantify viral RNA abundance in the cell samples, RT-qPCR was performed using the same primers employed for the supernatant viral load. *ACTB* (beta-actin) mRNA levels served as an internal control. RT-qPCR was performed using the iScript™ cDNA Synthesis Kit and iTaq Universal SYBR Green Supermix (Bio-Rad) and run on a Biorad CFX384 real-time system (C1000 Touch Thermal Cycler), according to the manufacturer’s instructions. The relative viral RNA abundance was normalized to the *ACTB* internal control. The relative viral RNA abundance of each treatment was normalized to NT samples.

### Prophylactic administration of Cas13d prior to coronavirus infection

For 229E virus challenges, MRC-5 cells seeded at 25,000 cells/well in 48-well plates, 50,000 cells/well in 24-well plates, or 150,000 cells/well in 12-well plates were transduced with 53.6 μl of lentivirus of Cas13d and 53.6 μl of lentivirus of guide per 100,000 cells. For the SARS-CoV-2 challenges, Vero E6 cells stably expressing NLS-Cas13d (referred to Vero E6/NLS-Cas13d) were seeded at 100,000 cells/well in 12-well plates and transduced with 53.6 μl of crRNA lentivirus per 100,000 cells. Two days later, the cells were washed once with PBS and infected with 229E (MRC-5) or SARS-CoV-2 (Vero E6) at the indicated MOI. After one hour of incubation, the inoculum was removed and cells were washed once with PBS (pH 7.4) followed by the addition of fresh DMEM media supplemented with 10% FBS. The supernatant was collected at 24, 48, and 72 hpi. The virus titer was determined by RT-qPCR.

### Cell viability assessment using the Incucyte live-cell imaging system

MRC-5 cells were transduced with Cas13d and crRNA components and challenged with 229E. The cell culture media included 100 nM of Cytotox Green Dye (#4633, IncuCyte) for the detection of dead cells. The plate was scanned once every 3 h with nine images per well for 5 days using the incuctye live-cell imaging system (Ensen Bioscience). The amount of cell death was quantified using the total integrated intensity of the green fluorescence as exported using the incucyte software (IncuCyte 2019B Rev2).

### Combination of Cas13d treatment with small-molecule antivirals for prophylaxis

The cytotoxicity and efficacy of the selected small-molecule drugs were first determined before performing combination assays with Cas13d/crRNA. MRC-5 or Vero E6/NLS-Cas13d cells were transduced with Cas13d/crRNA or crRNA components, respectively, as described above. Two days later, the cells were pre-treated with indicated drug for 1 h, and then infected with 229E or SARS-CoV-2 at an MOI of 0.01. After one hour of absorption, the inoculum was removed and the cells were washed once with PBS. Media containing the indicated drug was added. The supernatant was collected at 24 and 48 hpi and the virus titer was determined by RT-qPCR.

### RNA FISH assay

RNA FISH was performed following a previously published protocol^68^. Briefly, a set of FISH probes targeting 229E viral RNA was designed and ordered from IDT and labeled with AF674 (Thermo Scientific) following our previous work^69^. The cells were cultured in 8-well μ-slides (ibidi Cat# 80824). Cells were washed with PBS, fixed with 3.7% formaldehyde in PBS at room temperature for 10 min, washed twice with PBS, and then permeabilized with 70% (vol/vol) ethanol for >1 h at 4°C. After decanting 70% ethanol from the wells, 200 μL of Wash Buffer A (40 μL Stellaris® RNA FISH Wash Buffer A (Cat# SMF-WA1–60, LGC Biosearch Technologies), 20 μL of deionized formamide, 140 μL distilled nuclease-free H_2_O) was added to cells and incubated at room temperature for 5 min. After decanting Wash Buffer A, 100 μL of Hybridization Buffer (90 μL Stellaris^®^ RNA FISH Hybridization Buffer (Cat# SMF-HB1–10, LGC Biosearch Technologies), 10 μL of deionized formamide) containing 2 μL of 12.5 μM RNA FISH probes was added into each well and incubated for 16 h at 37 °C in the dark. Then cells were washed twice with Wash Buffer A by incubating for 30 min at 37°C in the dark and stored in Wash Buffer B (Cat# SMF-WA1–60, LGC Biosearch Technologies) for imaging.

### RNA FISH microscopy analysis

The cells were scanned in the z-axis of the imaging objective with a slice interval of 0.3 μm over a 1.2 μm range. The maximum projection of the z stack was created by Fiji (ImageJ)^70^ and used for quantification for all the signal channels. Cell outlines were generated from the ER signal channel. For each cell, the mean intensity of the FISH signal and Cas13d signal within the cytosol of each cell was measured by Fiji. The intensity was further corrected by subtracting the background of the same color channel.

### Confocal imaging of Cas13d and crRNAs

MRC-5 cells were cultured in poly-L-lysine pre-treated 8-well μ-slides (ibidi Cat# 80824). Cells were transduced with mCherry-fused NLS- or NES-Cas13d. One day after transduction, the cells were transfected with a Cy5-labeled crRNA (Synthego), 1 pmol per well, using the lipitoid delivery reagent following the procedure as described below. 4 h later, the cells were washed with DPBS twice, fixed with 3.7% formaldehyde in PBS at room temperature for 10 min, washed twice with DPBS, and stored in DPBS for imaging.

### Spinning disk confocal microscopy

Confocal microscopy was performed on a Nikon TiE inverted spinning disk confocal microscope (SDCM) equipped with a Photometrics Prime 95B camera, a CSU-X1 confocal scanner unit with microlenses, and 405 nm, 488 nm, 561 nm, and 642-nm lasers, using the 60 3 PLAN APO IR water objective (NA = 1.27). Images were taken using NIS Elements version 4.60 software with Z stacks at 0.3-mm steps. The camera pixel size of SDCM is 0.183 mm/pixel. The pinhole size is 50 mm. Only one Z slice is used for all images shown.

### Lipitoid transfection

Lipitoid and RNA complexes were prepared by combining RNA and 0.5 mM lipitoid solution prepared in distilled water. The solutions were warmed to room temperature prior to complex formation. As a sample calculation, for the generation of lipid nanoparticle (LNP) at 2/1 (positive/negative) charge ratio, 2.5 μg RNA was diluted in 47.5 μL OptiMEM, and in a separate tube, 7 μL 0.5 mM lipitoid was diluted in 43 μL OptiMEM. The two solutions were mixed by gentle aspiration, resulting in a complex formation solution. Following incubation for 10 min at room temperature with occasional agitation, the resulting LNP complexes were used immediately for transfection.

MRC cells were seeded at 120,000 cells/well in 12-well plates or at 65,000 cells/well in 24-well plates. Vero E6 cells were seeded at 100,000 cells/well in 24-well plates. One day later, cells were sequentially washed twice with PBS and once with OptiMEM. Cells were incubated in 0.9 mL of OptiMEM for 30 min. Lipitoid and mRNA complexes were prepared using 35 μL of 0.5 mM lipitoid and 7.5 μg of RNA as described above, and added to the cells at 100 µl/well. After 1 hr of incubation, 250 µl of 50% FBS was added to each well, and cells were incubated until the assay for transgene expression or for use in virus challenge assays. The transfection efficiency was analyzed by flow cytometry.

### Cas13d used as a treatment of coronavirus infection

For treatment of 229E infection, MRC-5 cells were seeded at 65,000 cells/well in 24-well plates. One day later, cells were infected with 229E at an MOI of 0.01. After one hour of absorption, the virus inoculum was removed and washed once with PBS. The cells were then transfected per well with 1.0 μg of mRNA encoding Cas13d (Trilink) and 0.8 μg of crRNA (Synthego) using Lipofectamine MessengerMAX (Invitrogen) at 0, 1, 3, and 6 hpi. The media was changed 6 h after transfection. The supernatant was collected at 48 hpi and viral RNA was isolated by using Quick-RNA Viral Kits (Zymo Research). The virus titer was determined by RT-qPCR. At 72 hpi, the media was removed and the cells were washed with PBS once, and then stained with crystal violet solution (1% crystal violet in 25% methanol and distilled H_2_O) for 15 min at room temperature. The plates were washed with distilled H_2_O three times and air-dried. The live-cell area was measured by analyzing photos of the stained wells with ImageJ^70^.

For treatment of SARS-CoV-2 infection, Vero E6 cells (stably expressing NES-Cas13d) were seeded at 100,000 cells/well in 24-well plates. The cells were infected with SARS-CoV-2 at an MOI of 0.01. The cells were washed with DPBS once after a one-hour inoculation. At 6 hpi, the cells were transfected with 0.8 μg per well of crRNA using the lipitoid delivery reagent as described above. Small-molecule drug was added 20 min after transfection. At 48 hpi, the virus titer in the supernatant was determined by RT-qPCR. The virus titer of some samples was also determined by plaque assay.

### Air–liquid interface (ALI) culture

Human primary bronchial epithelial cells (hPBEC) were transduced with NES-Cas13d using IAV- or VSV-pseudotyped lentivirus and cultured in PneumaCult-Ex Plus Medium (StemCell, Canada) supplemented with Hydrocortisone (0.5 μg/ml). After reaching 60–70% confluency, cells were transferred to 12- or 24-well plates and plated at 100,000 or 33,000 cells/insert using the Costar® 6.5 mm Transwell^®^, 0.4-µm Pore Polyester Membrane Inserts (StemCell, Canada). After 2–3 days, cells were lifted by removing the culture from the apical chamber and PneumaCult-ALI media (StemCell, Canada) was added to the basal chamber only. Differentiation into a pseudostratified mucociliary epithelium was achieved after ~21–27 days^49^.

Before being used for testing the Cas13d antiviral effect, the crRNA transfection efficiency in ALI cultures using LNP was tested by using the CytoFLEX Flow Cytometry (Beckman Coulter). Immediately before infection, the apical surfaces were washed twice to remove accumulated mucus with 500 µl of PBS at 37 °C with each wash lasting 30 min. ALI cell cultures were infected with 229E at an MOI of 0.05, with SARS-CoV-2 WA1 at an MOI of 0.6, or with SARS-CoV-2 Omicron variant at an MOI of 0.1 in 100 µl of medium for 1 h at 37 °C. The ALI cultures were transfected with crRNA using the LNP at 6 hpi. The LNP complex was added to the apical surface of the ALI cultures for 60 min and then removed followed by a single wash with PBS. At 48 and 72 hpi, the cell apical surface was washed with 100 µl of PBS to collect the released virus. The viral RNA was isolated and the virus titer was determined by RT-qPCR.

### RNA-seq for evaluation of off-target effects on the transcriptome

For specificity analysis of Cas13d off-target effects, RNA-seq was performed on mRNA from transfection experiments. The lipitoid delivery reagent was used to transfect Cas13d mRNA (Trilink) and crRNAs (Synthego) as described above. 48 h post-transfection, total RNA was collected and isolated using a Qiagen RNeasy Plus Mini kit. mRNA was then extracted using a NEBNext Poly(A) mRNA Magnetic Isolation Module and RNA- seq libraries were prepared using a NEBNext Ultra Directional RNA Library Prep Kit for Illumina sequencing. RNA-seq libraries were sequenced on an Illumina NextSeq instrument with at least 30 million reads per library.

STAR 2.5.4b was used to generate a genome index using hg19 and sjdbOverhang set to 100^71^. Paired-end reads were aligned to the genome using STAR 2.5.4b. HTSeq-count was used to determine read counts^72^. Fragments per kilobase of transcript per million mapped reads (FPKM) for each gene were determined by dividing the read count by the gene length in kb and by the total read count in millions.

### Conservation analysis of the target site of the SARS-CoV-2 crRNA SN1

We retrieved sequences of 9,692,670 SARS-CoV-2 isolates from the Global Initiative on Sharing All Influenza Data (GISAID) database (as of 03/16/2022)^27^. Bowtie was used to determine whether SN1 is in perfect alignment to any sequence of 2,473,889 SARS-CoV-2 isolates^73^. The specific variants listed in Fig. 1g are USA-WA1/2020 MN985325, Alpha MZ344997.1, Beta MW598419.1, Delta MZ359841.1, Epsilon MW453103.1, Gamma MZ169911.1, Zeta MW988205, Eta EPI_ISL_2188489 [https://www.epicov.org/epi3/frontend#dc454], Kappa EPI_ISL_3843727 [https://www.epicov.org/epi3/frontend#dc454], Lambda EPI_ISL_3023583 [https://www.epicov.org/epi3/frontend#dc454], Iota EPI_ISL_2776162 [https://www.epicov.org/epi3/frontend#dc454], Mu EPI_ISL_3385846 [https://www.epicov.org/epi3/frontend#dc454], Omicron EPI_ISL_7160424 [https://www.epicov.org/epi3/frontend#dc454].

### IAV-pseudotyped lentivirus generation and transduction

On day 1, a 10-cm plate of HEK293T cells was trypsinized with 3 mL 0.05% trypsin and resuspended in 6 mL of fresh DMEM medium until monodispersed. A 1.8 mL aliquot of monodispersed HEK293T cells was plated in a new 10 cm dish. On day 2, transfection complexes were made by mixing the following in a 2-mL tube: 1.5 mL opti-MEM media, 5.31 µg psPAX2, 5.31 µg pI.18-HA, 5.31 µg transfer plasmid ps, 1.05 µg pCAGGS-HAT, and 0.531 µg pCI-M2. Mirus LT1 reagent (52.5 µL) was gradually added with mixing and the transfection mixture was incubated at room temperature for 30 min. The mixture was then added dropwise to the HEK293T cells. On day 3, media was removed from transfected cells and replaced with fresh DMEM media. ViralBoost reagent and recombinant *Clostridium perfringens* neuraminidase were added for a final concentration of 1x and 1.0 units/ml, respectively. On day 4, the cell culture supernatant was collected and filtered through a 0.45-µm membrane. Lentivirus precipitation solution was added for a final concentration of 1× and the solution was stored overnight at 4 °C overnight. Precipitated lentivirus was collected by centrifugation (1500×*g* for 30 min at 4 °C) with the pellet resuspended in fresh DMEM.

### Data analysis

Data visualizations (graphs) were performed in GraphPad Prism software version 9. All the flow cytometry data has been analyzed using FlowJo v10. An example of the gating strategy is shown in Supplementary Fig. 8g. *P* values were calculated in Excel. Statistical analyses were performed using a two-sided *t* test with equal variance for all RT-qPCR data, using a two-sided *t* test with unequal variance for RNA FISH data (Fig. 3c–e), and using a one-sided *t* test with equal variance for plaque assay data (Fig. 5b). *P* values for all figures are listed in Supplementary Data 3. A *P* value <0.05 is considered statistically significant.

### Reporting summary

Further information on research design is available in the Nature Research Reporting Summary linked to this article.

## Supplementary information

Supplementary Information

Reporting Summary

Description of Additional Supplementary Files

Supplementary Movie 1

Supplementary Movie 2

Supplementary Movie 3

Supplementary Movie 4

Supplementary Data 1

Supplementary Data 2

Supplementary Data 3

## Source data

Source Data

## Data Availability

The RNA sequencing data generated in this study have been deposited in the GEO database under accession number GSE186020. The key plasmids constructed in this study, their sequences, and maps have been deposited to Addgene (# 155305, # 155307).  Source data are provided with this paper.
