# Supplementary material for: Broad-spectrum CRISPR-mediated inhibition of SARS-CoV-2 variants and endemic coronaviruses in vitro
Source: Nat Commun. 2022 May 19;13:2766. doi: 10.1038/s41467-022-30546-7 (PMC9119983; doi:10.1038/s41467-022-30546-7)
Supplement: Supplementary file 1 — Supplementary Information [file 41467_2022_30546_MOESM1_ESM.pdf]

Broad-spectrum CRISPR-mediated inhibition of SARS-CoV-2 variants and endemic coronaviruses in vitro

Leiping Zeng[1]*, Yanxia Liu[1]*, Xammy Huu Nguyenla[2], Timothy R. Abbott[1], Mengting Han[1], Yanyu Zhu[1], Augustine Chemparathy[1], Xueqiu Lin[1], Xinyi Chen[1], Haifeng Wang[1], Draven A. Rane[1], Jordan M. Spatz[3], Saket Jain[4], Arjun Rustagi[5], Benjamin Pinsky[5,6], Adrianna E. Zepeda[7], Anastasia P. Kadina[7], John A. Walker III[7], Kevin Holden[7], Nigel Temperton[8], Jennifer R. Cochran[1], Annelise E. Barron[1], Michael D. Connolly[9], Catherine A. Blish[5,11], David B. Lewis[3], Sarah A. Stanley[2]†, Marie F. La Russa[1]†, Lei S Qi[1,10,11]†

Affiliations: [1]Department of Bioengineering, Stanford University, Stanford, CA 94305; [2]Department of Molecular and Cellular Biology, University of California, Berkeley, CA 94720; [3]Department of Pediatrics, Stanford University, Stanford, CA 94305; [4]University of California San Francisco, San Francisco, CA 94143; [5]Department of Medicine, Stanford University, Stanford, CA 94305; [6]Department of Pathology, Stanford University, Stanford CA; [7]Synthego Corporation, Redwood City, CA; [8]Viral Pseudotype Unit, Medway School of Pharmacy, Chatham, Kent ME4 4TB, United Kingdom; [9]Lawrence Berkeley National Laboratory, Berkeley, CA 94720; [10]ChEM-H, Stanford University, Stanford, CA 94305; [11]Chan Zuckerberg BioHub, San Francisco, CA 94158.

*These authors made equal contributions to the work.

† Correspondence: S.A.S. (sastanley@berkeley.edu), M.F.L. (mlarussa@stanford.edu), L.S.Q. (stanley.qi@stanford.edu)

Keywords: broad-spectrum antiviral, CRISPR-Cas13, PAC-MAN, antiviral, SARS-CoV-2, COVID-19, 229E, OC43, antiviral drugs, treatment

Supplementary Figures

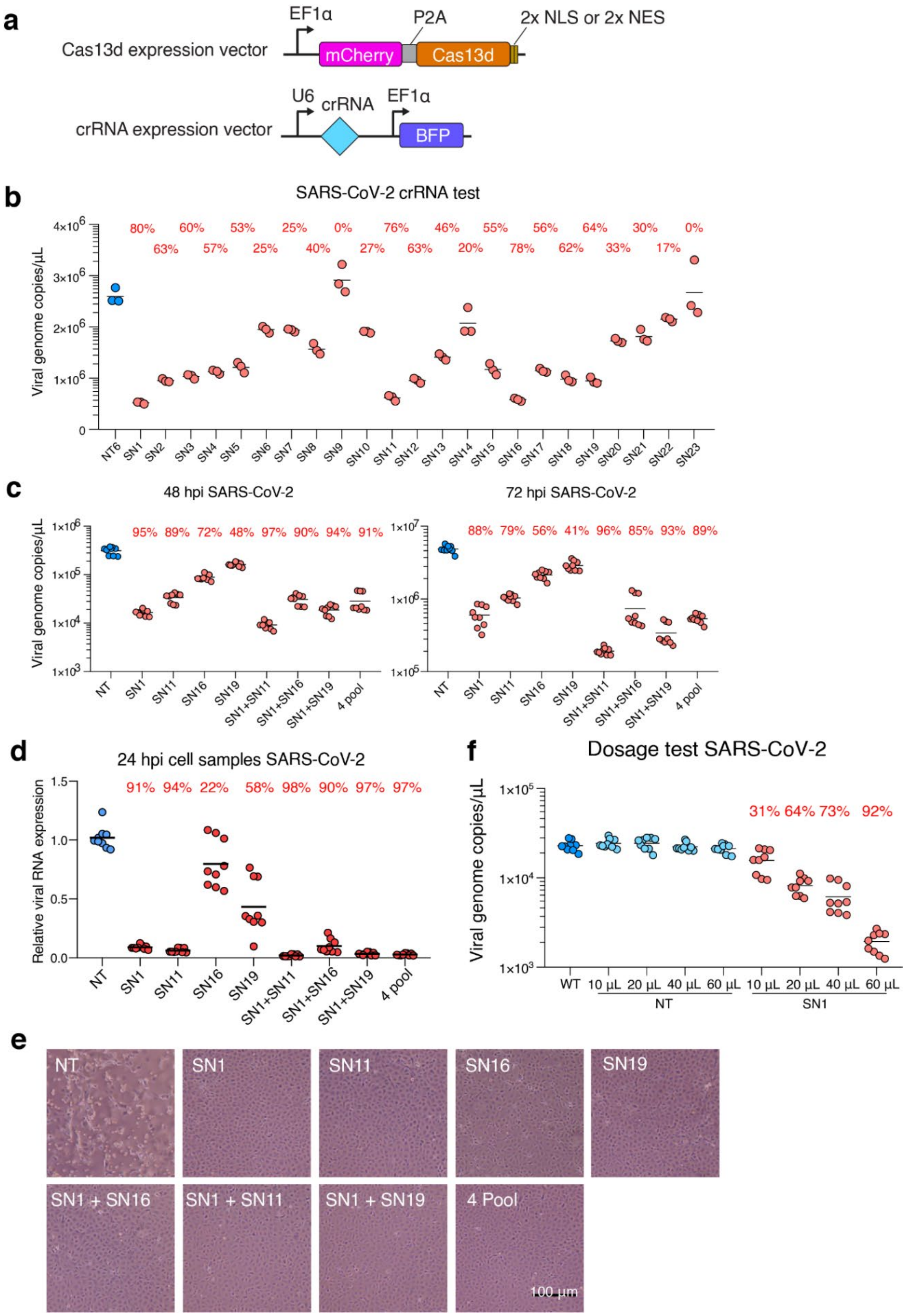

Supplementary Figure 1 | Cas13d inhibits SARS-CoV-2 virus. (a) The expression vector of Cas13d and crRNA used in this study. **(b)** Viral genome copies determined by RT-qPCR. The 23 crRNAs targeting the N gene of SARS-CoV-2 were tested in Vero E6/Cas13d stably transduced cells. NT, non-targeting crRNA. Cells were challenged with the USA-WA1/2020

strain of SARS-CoV-2; n = 1, t = 3. **(c)** The virus titer of the supernatant collected at 48 and 72 hpi. The best crRNAs from (b) were tested singly or combined as indicated; n = 3, t = 3. **(d)** The cell lysates of the same experiment from Figure 1e were collected at 24hpi and RT-qPCR was performed to quantify the relative expression of the N gene; n = 3, t = 3. **(e)** Brightfield images of Vero E6/NLS-Cas13d cells expressing indicated crRNA(s) and challenged with SARS-CoV-2; n = 1. **(f)** The virus production from cells that were transduced with different doses of lentivirus expressing the NT or SN1 crRNAs; n = 3, t = 3. n is the number of independent biological experiments. t is the number of technician replicates per biological replicate in the RT-qPCR assay. All source data in this figure are provided as a Source data file. p values are listed in Supplementary Data 3.

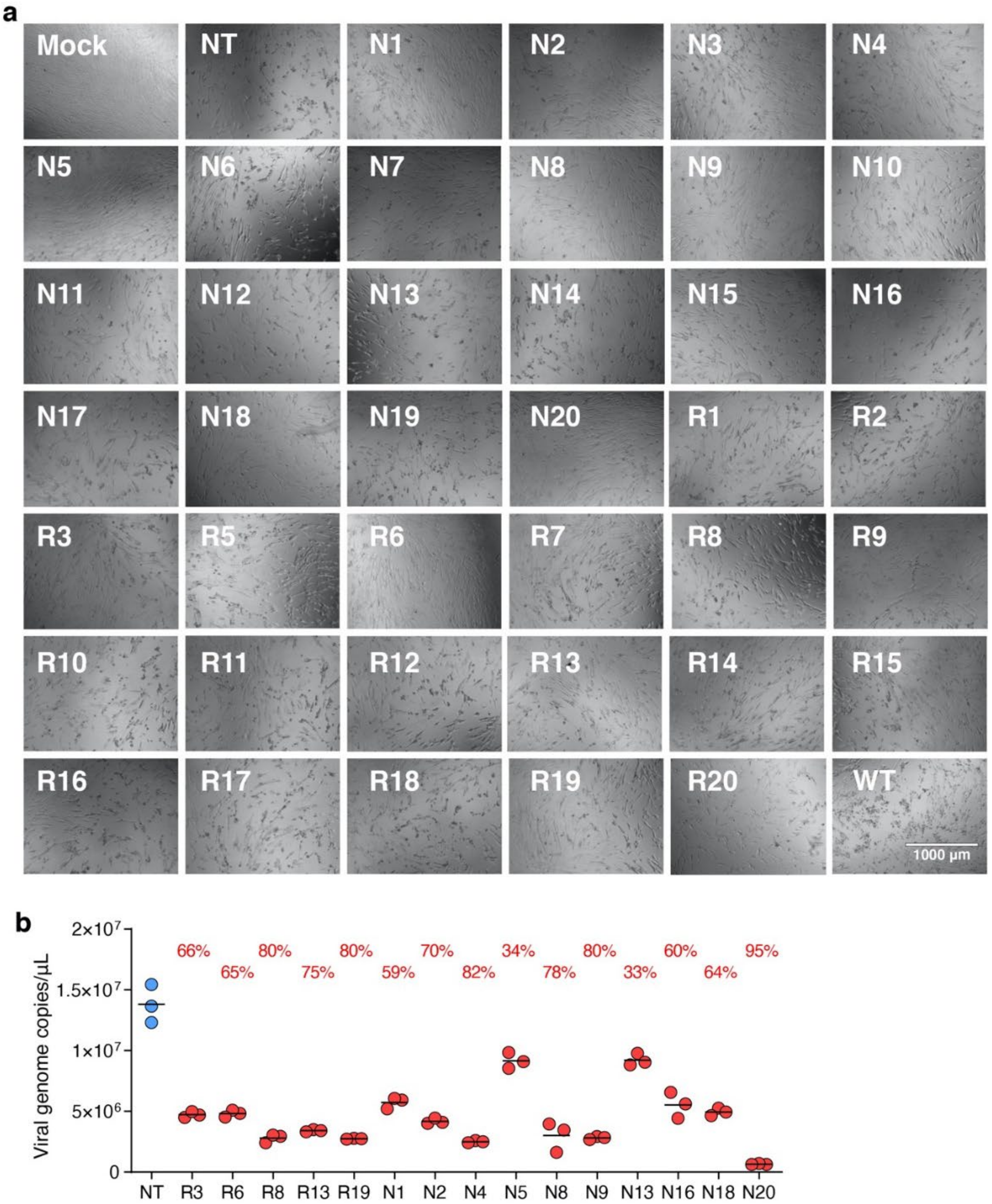

Supplementary Figure 2 | Cas13d inhibits human coronavirus HCoV-229E. (a) Bright field images of MRC-5 cells after 229E infection at 72 hpi; n = 1. The cells were co-transduced with NLS Cas13d and the indicated crRNAs and challenged with 229E 2 days later. **(b)** Viral genome copies in supernatant collected at 48 hpi were determined by RT-qPCR; n = 1, t = 3. n is the number of independent biological experiments. t is the number of technician replicates per biological replicate in the RT-qPCR assay. All source data in this figure are provided as a Source data file. p values are listed in Supplementary Data 3.

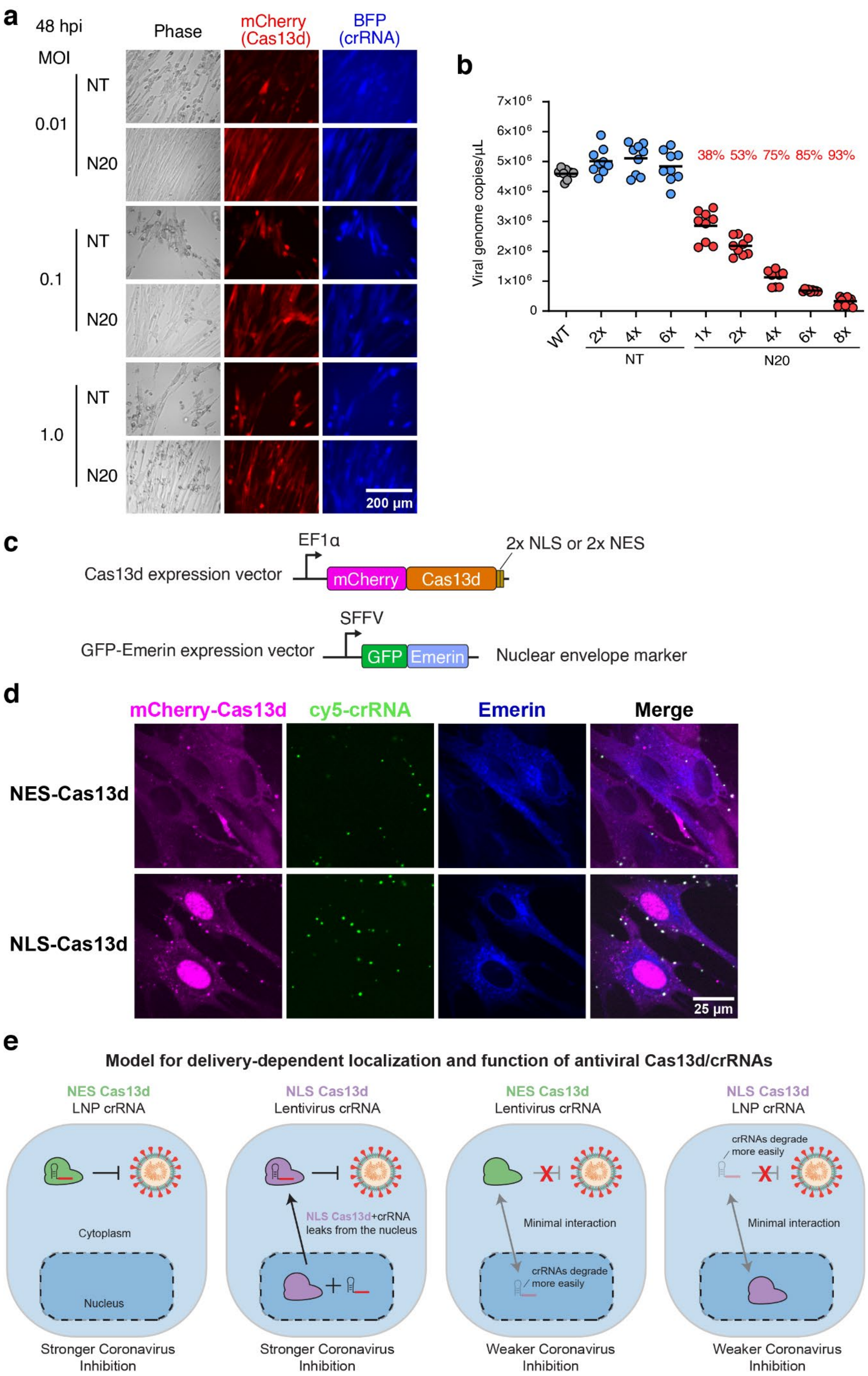
a
48 hpi
MOI
Phase
mCherry (Cas13d)
BFP (crRNA)
0.01
0.1
1.0
NT
N20
200 μm
b
Viral genome copies/μL
0
1×10⁶
2×10⁶
3×10⁶
4×10⁶
5×10⁶
6×10⁶
7×10⁶
38% 53% 75% 85% 93%
WT
2X
4X
6X
1X
8X
NT
N20
c
Cas13d expression vector
EF1α
mCherry
Cas13d
2x NLS or 2x NES
GFP-Emerin expression vector
SFFV
GFP
Emerin
Nuclear envelope marker
d
mCherry-Cas13d
cy5-crRNA
Emerin
Merge
NES-Cas13d
NLS-Cas13d
25 μm
e
Model for delivery-dependent localization and function of antiviral Cas13d/crRNAs
NES Cas13d
LNP crRNA
NLS Cas13d
Lentivirus crRNA
NES Cas13d
Lentivirus crRNA
NLS Cas13d
LNP crRNA
Cytoplasm
Nucleus
NLS Cas13d+crRNA leaks from the nucleus
Minimal interaction
crRNAs degrade more easily
Minimal interaction
crRNAs degrade more easily
Stronger Coronavirus Inhibition
Stronger Coronavirus Inhibition
Weaker Coronavirus Inhibition
Weaker Coronavirus Inhibition

Supplementary Figure 3 | Characterization of Cas13-mediated viral inhibition. (a) Microscopic images of MRC-5 cells infected with 229E at an MOI of 0.01, 0.1, or 1.0 at 48 hpi. NT, non-targeting crRNA; N20, 229E viral targeting crRNA; n = 3. **(b)** Virus production from cells that were transduced with different doses of lentiviruses of NLS Cas13d and either an NT crRNA or N20 crRNA; n = 3, t = 3. **(c)** The expression construct of mCherry infused Cas13d and the nuclear envelope marker GFP-Emerin were used for the **(d)** fluorescent microscopy of MRC-5 cells expressing mCherry-fused NES or NLS Cas13d along with GFP-Emerin (nuclear envelope marker), transfected Cy5 labeled crRNA, and fixed at 4 h post-transfection; n = 3. Punctae may indicate aggregates of Cas13d/crRNA without targeting RNAs. **(e)** Schema delineates that lentivirus and LNP delivered crRNA shows effective antiviral activity with NLS- and NES-Cas13d, respectively. n is the number of independent biological experiments. t is the number of technician replicates per biological replicate in the RT-qPCR assay. All source data in this figure are provided as a Source data file. p values are listed in Supplementary Data 3.

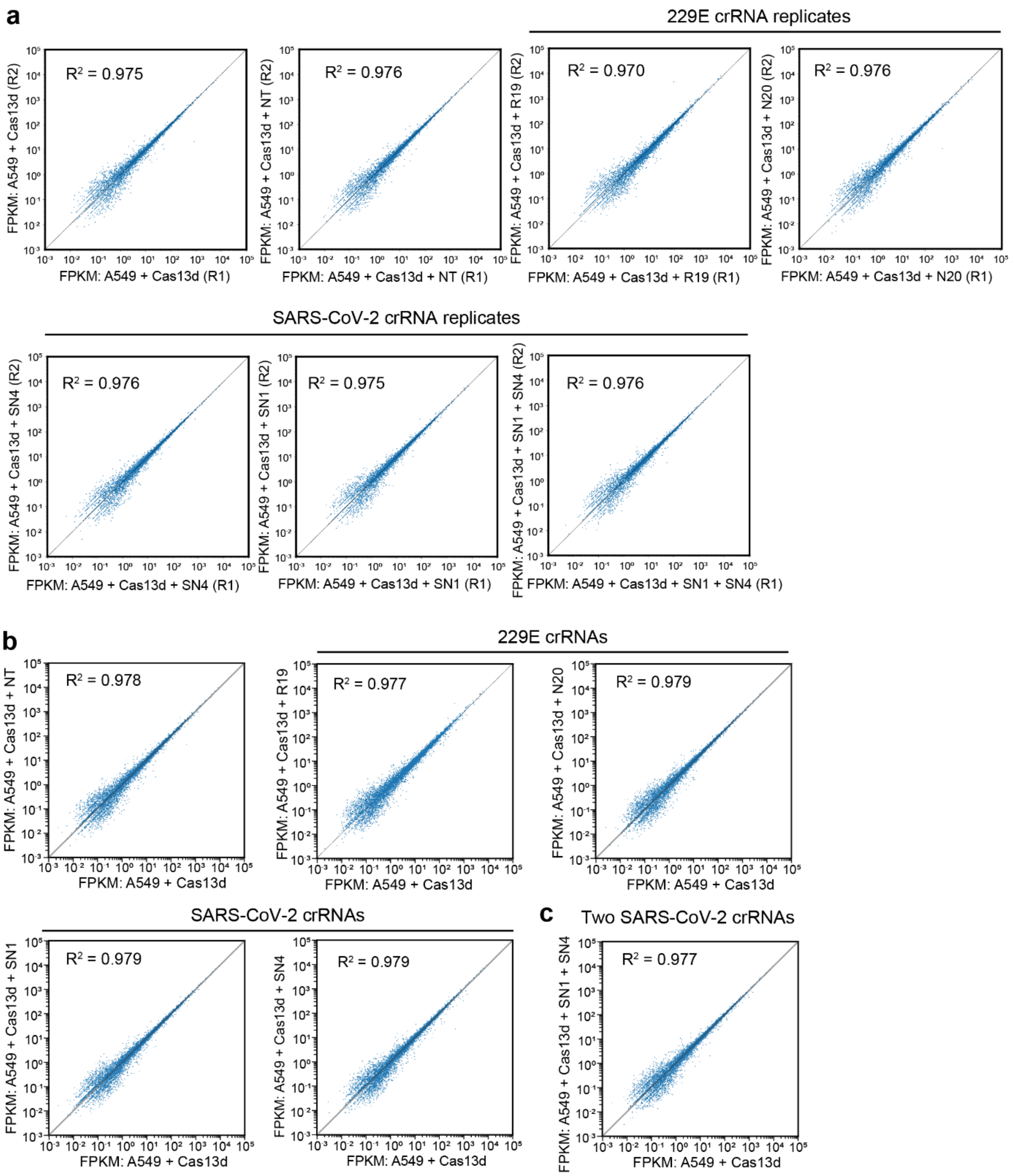

Supplementary Figure 4 | The SARS-CoV-2 and 229E virus-targeting crRNAs are highly specific. **(a)** Comparison of two replicates for each sample type of RNA-seq profiling data in A549 cells expressing Cas13d alone or Cas13d with one or two crRNAs without coronavirus targets. **(b)** RNA-seq profiling of A549 cells expressing Cas13d plus the non-targeting crRNA (NT), 229E crRNA R19, N20 or the SARS-CoV-2 crRNA SN1, SN4 compared with the cells only expressing Cas13d; n = 2 biological replicates. **(c)** The transcriptome of A549 cells expressing Cas13d and two crRNAs, SN1 and SN4, were compared with the cells expressing Cas13d only; n = 2 biological replicates.

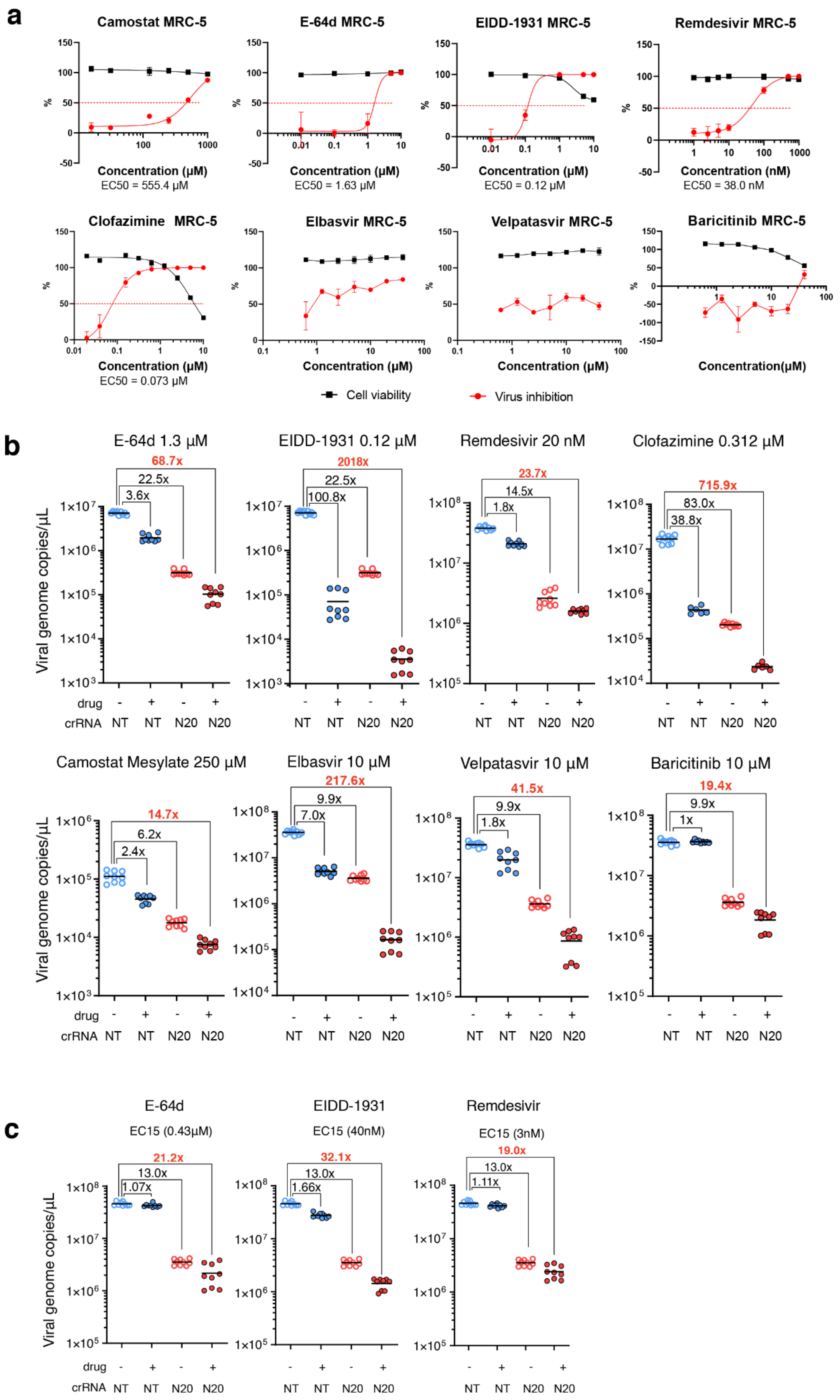

Supplementary Figure 5 | The combination of Cas13d antivirals with small molecule

antiviral drugs enhances viral inhibition. (a) The cytotoxicity and efficacy of indicated antiviral drugs in MRC-5 cells against 229E infection; for cytotoxicity test, n = 2; for virus inhibition efficacy test, n = 3. **(b-c)** Viral genome copies determined by RT-qPCR; n = 3, t = 3. **(b)** Cas13d and crRNA N20 were tested against 229E virus replication in combination with the indicated antiviral drugs at the indicated concentrations. **(c)** Cas13d and crRNAs combined with the indicated small molecule drugs at a lower dose (EC15). n is the number of independent biological experiments. t is the number of technician replicates per biological replicate in the RT-qPCR assay. All source data in this figure are provided as a Source data file. p values are listed in Supplementary Data 3.

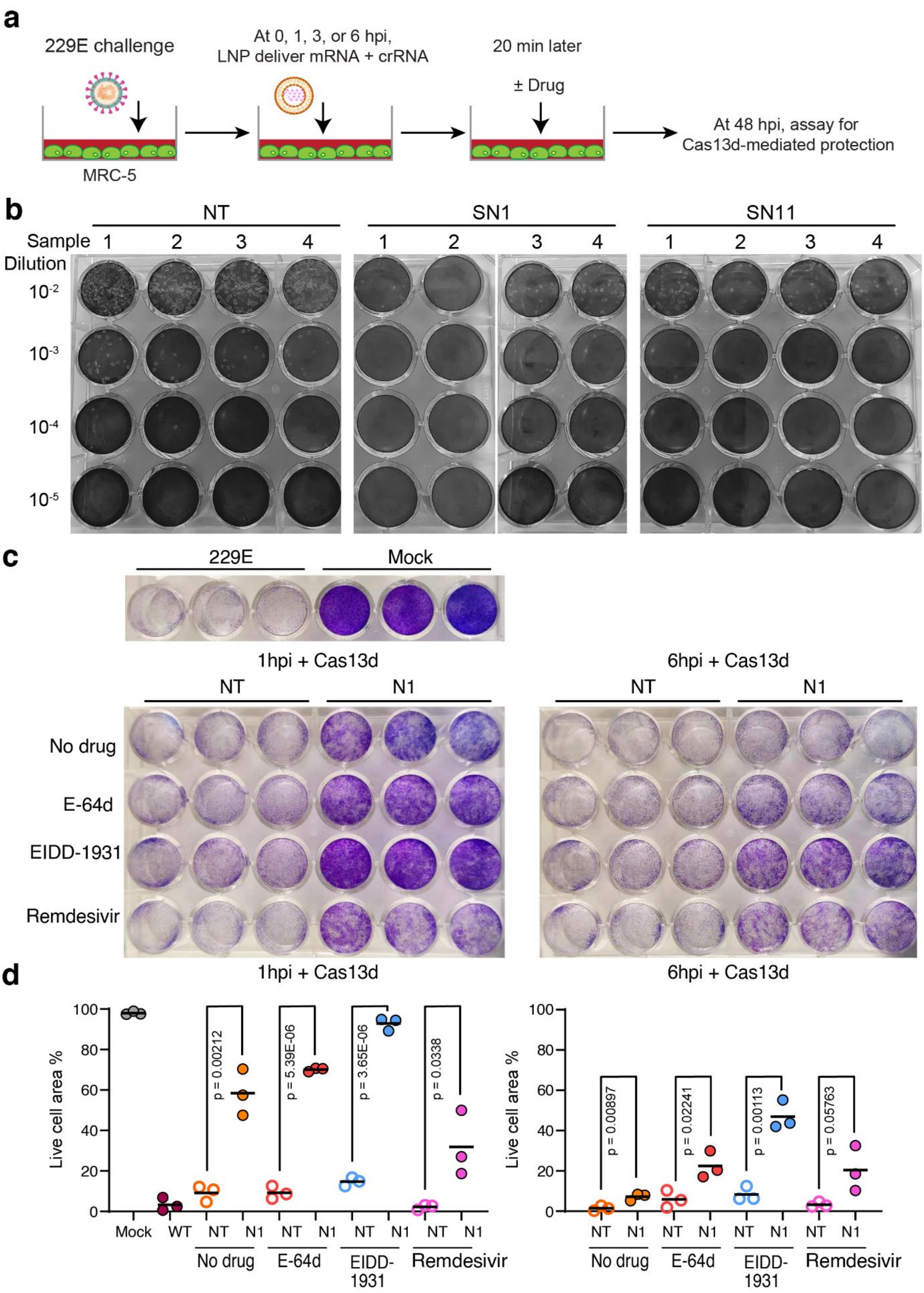

Supplementary Figure 6 | Cas13d and antiviral crRNAs are effective for treatment of established 229E and SARS-CoV-2 infection. (a) Schematic of the treatment of 229E infection. **(b)** Crystal violet staining of the Vero E6 cells at 5 days post-infection for plaque assay of the samples from **Figure 5b**. **(c)** Crystal violet staining of MRC-5 cells. MRC-5 cells

were mock-infected or infected with 229E at an MOI of 0.01. Cas13d mRNA and NT or N1 crRNA were delivered into the cells at 1 and 6 hpi by using Lipofectamine MessengerMax transfection. The cells were stained with crystal violet and photographed at 72 hpi. **(d)** Percent live-cell area of the stained cells was plotted; n = 3 wells. All source data in this figure are provided as a Source data file. p values are listed in Supplementary Data 3, calculated by two-tailed Student’s t-test.

Supplementary fig. 7

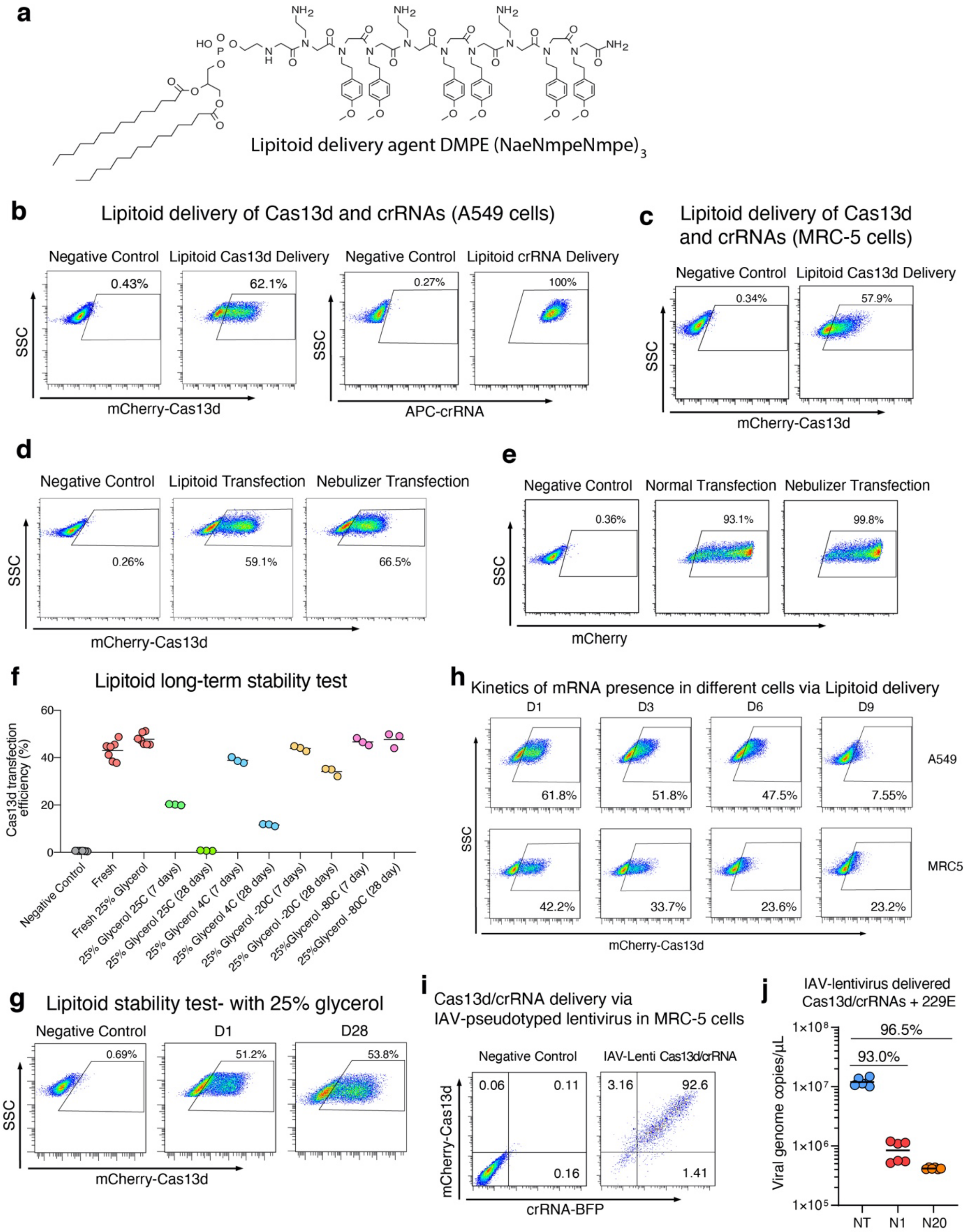

Supplementary Figure 7 | Characterization of lipitoid delivery of Cas13d and crRNAs. **(a)** Structure of the lipitoid delivery agent used in this study. **(b-c)** Flow cytometry analysis of A549 **(b)** and MRC5 **(c)** cells 2 days after transfection with Cas13d-mCherry mRNA and crRNA-cy5 using the lipitoid delivery agent. **(d-e)** Flow cytometry analysis of lipitoid delivery of mCherry-Cas13d **(d)** or mCherry **(e)** mRNA after standard transfection or transfection by nebulization. Delivery efficiency was determined by flow cytometric analysis 2 days after transfection. **(f)** The transfection efficiency of Cas13d-mCherry mRNA via lipitoid delivery; n =

6 for the negative control, fresh-made lipitoid complex, and fresh-made lipitoid complex containing 25% glycerol; n = 3 for the rest groups. Storage time is indicated in parentheses. The lipitoid nanoparticles supplemented with 25% Glycerol maintained their transfection capacity after 28 days of storage at -80°C. **(g)** Flow cytometric analysis of transfection with mCherry-Cas13d that had been stored in 25% glycerol for 1 or 28 days. **(h)** Flow cytometric analysis showing the kinetics of mCherry-Cas13d mRNA presence in A549 and MRC5 cells after lipitoid delivery. **(i)** The delivery efficiency of IAV-pseudotyped lentivirus containing mCherry-Cas13d or a crRNA and a BFP cassette in MRC-5 cells. **(j)** Viral genome copies of 229E determined by RT-qPCR. MRC-5 cells were co-transduced with NLS Cas13d and crRNA NT, N1, or N20 using IAV-pseudotyped lentivirus. The cells were infected with 229E 2 days later. Supernatant samples were collected and virus titer was determined at 48 hpi; n = 2, t = 3. p values for N1 ($p = 5.74\times10^{-8}$) and N20 ($p = 3.48\times10^{-8}$) were calculated relative to the viral titer obtained with the NT crRNA, by two-tailed Student's t-test. n is the number of independent biological experiments. t is the number of technician replicates per biological replicate in the RT-qPCR assay. All source data in this figure are provided as a Source data file.

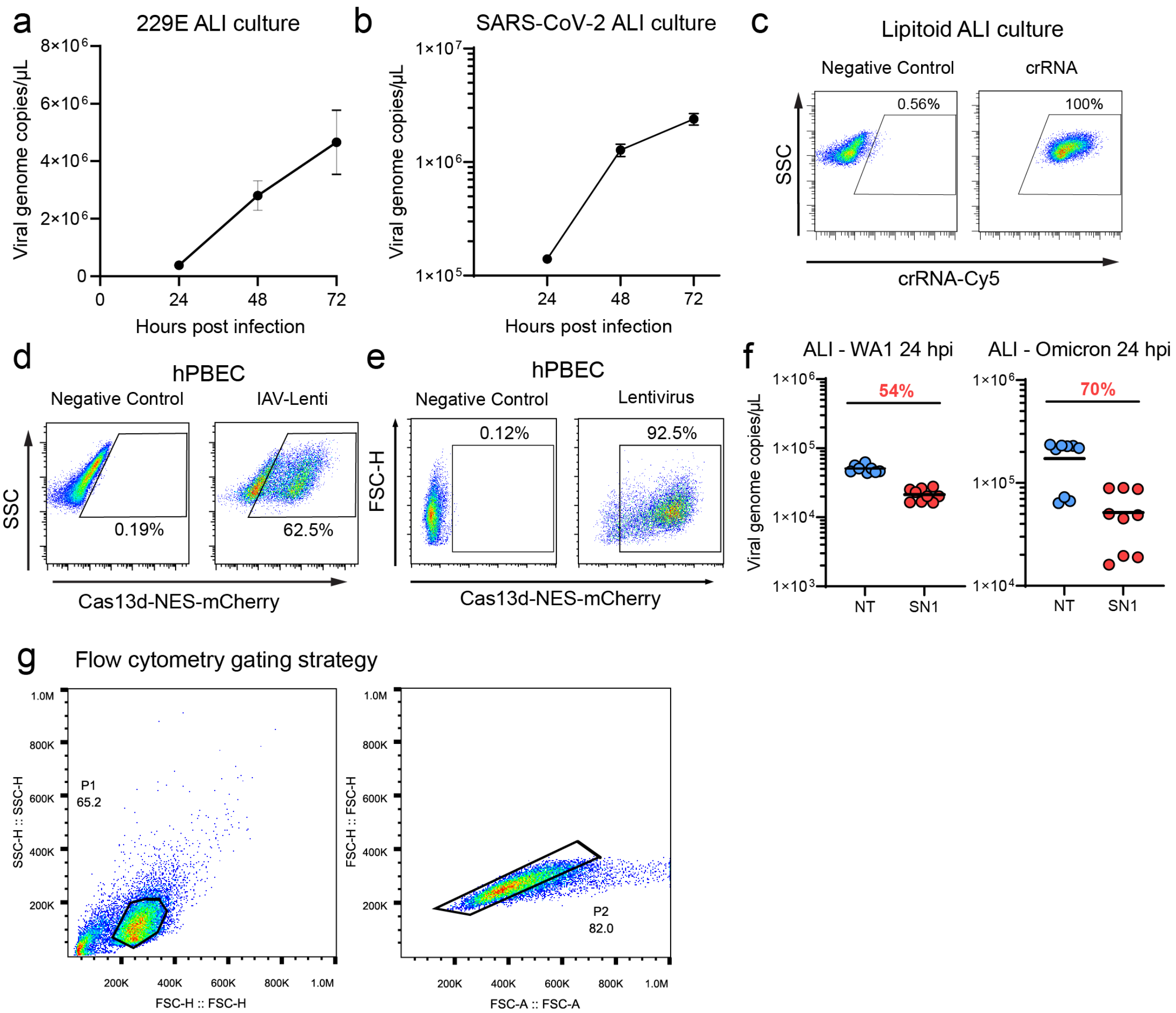

Supplementary Figure 8 | Growth curve of 229E coronavirus and delivery of crRNAs using the lipitoid delivery agent in ALI cultures. (a-b) The growth curve of 229E (at an MOI of 0.05) and SARS-CoV-2 (at an MOI of 0.6) virus in air-liquid interface (ALI) cultures; n = 3. Data presented as means ± SEM. **(c)** Flow cytometry analysis of crRNA (labeled with cy5) delivery using LNP in ALI cultures. **(d-e)** Flow cytometry of hPBECs delivered with NES Cas13d using IAV- or VSV-pseudotyped lentivirus. **(f)** The titer of SARS-CoV-2 virus, including WA1 and Omicron strains, was determined at 24 hpi; n = 3, t = 3. (g) The example of gating strategy for flow cytometry analysis. n is the number of independent biological experiments. t is the number of technician replicates per biological replicate in the RT-qPCR assay. All source data in this figure are provided as a Source data file. p values are listed in Supplementary Data 3.
