# Supplementary material for: Broad-spectrum CRISPR-mediated inhibition of SARS-CoV-2 variants and endemic coronaviruses in vitro
Source: Nat Commun. 2022 May 19;13:2766. doi: 10.1038/s41467-022-30546-7 (PMC9119983; doi:10.1038/s41467-022-30546-7)
Supplement: Supplementary file 2 — Reporting Summary [file 41467_2022_30546_MOESM2_ESM.pdf]

nature portfolio

Corresponding author(s): Lei S. Qi

Last updated by author(s): Apr 24, 2022

Reporting Summary

Nature Portfolio wishes to improve the reproducibility of the work that we publish. This form provides structure for consistency and transparency in reporting. For further information on Nature Portfolio policies, see our Editorial Policies and the Editorial Policy Checklist.

Statistics

For all statistical analyses, confirm that the following items are present in the figure legend, table legend, main text, or Methods section.

n/a	Confirmed	
☐	☒	The exact sample size (n) for each experimental group/condition, given as a discrete number and unit of measurement
☐	☒	A statement on whether measurements were taken from distinct samples or whether the same sample was measured repeatedly
☐	☒	The statistical test(s) used AND whether they are one- or two-sided<br>*Only common tests should be described solely by name; describe more complex techniques in the Methods section.*
☒	☐	A description of all covariates tested
☒	☐	A description of any assumptions or corrections, such as tests of normality and adjustment for multiple comparisons
☐	☒	A full description of the statistical parameters including central tendency (e.g. means) or other basic estimates (e.g. regression coefficient) AND variation (e.g. standard deviation) or associated estimates of uncertainty (e.g. confidence intervals)
☐	☒	For null hypothesis testing, the test statistic (e.g. F, t, r) with confidence intervals, effect sizes, degrees of freedom and P value noted<br>*Give P values as exact values whenever suitable.*
☒	☐	For Bayesian analysis, information on the choice of priors and Markov chain Monte Carlo settings
☒	☐	For hierarchical and complex designs, identification of the appropriate level for tests and full reporting of outcomes
☐	☒	Estimates of effect sizes (e.g. Cohen's d, Pearson's r), indicating how they were calculated

Our web collection on statistics for biologists contains articles on many of the points above.

Software and code

Policy information about availability of computer code

Data collection	No software was used for data collection.
Data analysis	ImageJ 1.53c<br>STAR 2.5.4b<br>HTSeq 0.13.5<br>FlowJo v10<br>GraphPad Prism v09<br>Bowtie 1.3.1<br>Cas13design v0.2 https://gitlab.com/sanjanalab/cas13/-/tree/master/Cas13designGuidePredictor

For manuscripts utilizing custom algorithms or software that are central to the research but not yet described in published literature, software must be made available to editors and reviewers. We strongly encourage code deposition in a community repository (e.g. GitHub). See the Nature Portfolio guidelines for submitting code & software for further information.

Data

Policy information about availability of data

All manuscripts must include a data availability statement. This statement should provide the following information, where applicable:

- Accession codes, unique identifiers, or web links for publicly available datasets
- A description of any restrictions on data availability
- For clinical datasets or third party data, please ensure that the statement adheres to our policy

All the databases/datasets used in the study are available in the manuscript under the “Data availability” section as well as in this reporting summary. All the raw data supporting the findings are available in the Source Data file submitted with this manuscript. The RNA-seq raw data is available in the GEO database under accession number GSE186020. Human reference genome Hg19 is used for analyzing the transcriptome using the RNA-seq data.

Field-specific reporting

Please select the one below that is the best fit for your research. If you are not sure, read the appropriate sections before making your selection.

☒ Life sciences ☐ Behavioural & social sciences ☐ Ecological, evolutionary & environmental sciences

For a reference copy of the document with all sections, see nature.com/documents/nr-reporting-summary-flat.pdf

Life sciences study design

All studies must disclose on these points even when the disclosure is negative.

Sample size	The sample sizes were determined to match the standards in comparable studies available in the literature (Timothy R. Abbott et al, Cell, 2020).
Data exclusions	Experiments were optimized in pilot assays before generating high-quality publication data. No data was excluded from the analysis.
Replication	All experiments were repeated at least 3 times as biological replicates with following exceptions:<br>- The data in Figure 2h was performed with two biological replicates due to large number of samples were collected for screening of the best effective crRNAs targeting the human coronavirus OC43.<br>- The data in Supplementary Figure 1b and Supplementary Figure 2b were performed with one biological replicate due to large number of samples were collected for a pilot screening of the best effective crRNAs.<br>- The cytotoxicity data in Supplementary Figure 5a were performed with two biological replicates since two replicates are enough for determination of drug toxicity.<br>- The data in Supplementary 7j was performed with two biological replicates and three technician replicates per biological replicate.<br><br>All the replication attempts were successful.
Randomization	No randomization was used in this study. Due to the small sample randomization was not relevant for this study. Covariates were controlled for by running control (non targeting crRNAs) every time.
Blinding	No blinding was used in this study. RNA targeting with Cas13 has been well-established in the field by independent groups using assays that do not require blinding.

Reporting for specific materials, systems and methods

We require information from authors about some types of materials, experimental systems and methods used in many studies. Here, indicate whether each material, system or method listed is relevant to your study. If you are not sure if a list item applies to your research, read the appropriate section before selecting a response.

Materials & experimental systems

n/a	Involved in the study	
☒	☐	Antibodies
☐	☒	Eukaryotic cell lines
☒	☐	Palaeontology and archaeology
☒	☐	Animals and other organisms
☒	☐	Human research participants
☒	☐	Clinical data
☒	☐	Dual use research of concern

Methods

n/a	Involved in the study	
☒	☐	ChIP-seq
☐	☒	Flow cytometry
☒	☐	MRI-based neuroimaging

Eukaryotic cell lines

Policy information about cell lines

Cell line source(s)	HEK 293T (ATCC CRL-3216); A549 (ATCC CCL-185); Vero E6 (ATCC CRL-1586); MRC-5 (ATCC CCL-171); Human primary bronchial epithelial cells (HBECs) (ATCC PCS300010)
Authentication	Cell lines were authenticated by the supplier ATCC. We did not perform any additional authentication upon reception. We made bulk stocks for each cell line after recovering from the original frozen vials. We discard the cells after passage for 40 days and thaw new cells from liquid nitrogen stocks. Cell morphology was monitored at each passage by microscope.
Mycoplasma contamination	Cells were routinely tested (PCR based test) and were mycoplasma negative.
Commonly misidentified lines (See ICLAC register)	No commonly misidentified cell lines were used in this manuscript.

Flow Cytometry

Plots

Confirm that:

- [x] The axis labels state the marker and fluorochrome used (e.g. CD4-FITC).
- [x] The axis scales are clearly visible. Include numbers along axes only for bottom left plot of group (a 'group' is an analysis of identical markers).
- [x] All plots are contour plots with outliers or pseudocolor plots.
- [x] A numerical value for number of cells or percentage (with statistics) is provided.

Methodology

Sample preparation	For measuring cell transduction efficiency, cells were re-suspended in 300 μL of 1x PBS containing 2% FBS and 0.5 mM EDTA for flow cytometry analysis.
Instrument	All samples were analyzed by an CytoFLEX Flow Cytometer (Beckman Coulter).
Software	All flow cytometry profiles were analyzed using FlowJo v10 software (Tree Star Inc).
Cell population abundance	No sorting was used in this study.
Gating strategy	Single cells were gated for all analysis. Positive cells were indicated by gates according to negative control samples.

- [x] Tick this box to confirm that a figure exemplifying the gating strategy is provided in the Supplementary Information.
