# Supplementary material for: Broad-spectrum CRISPR-mediated inhibition of SARS-CoV-2 variants and endemic coronaviruses in vitro
Source: Nat Commun. 2022 May 19;13:2766. doi: 10.1038/s41467-022-30546-7 (PMC9119983; doi:10.1038/s41467-022-30546-7)
Supplement: Supplementary file 3 — Description of Additional Supplementary Files [file 41467_2022_30546_MOESM3_ESM.pdf]

Title: Supplementary Movie 1
Description: Time-lapse microscope imaging of MRC5 cells without 229E infection. Green signal represents dead cells stained by Cytotox Green Dye (#4633, Incucyte).

Title: Supplementary Movie 2
Description: Time-lapse microscope imaging of MRC5 cells with 229E infection at an MOI of 0.01. Green signal represents dead cells stained by Cytotox Green Dye (#4633, Incucyte).

Title: Supplementary Movie 3
Description: Time-lapse microscope imaging of MRC5 cells transduced with NLS-Cas13d and crRNA NT and infected with 229E at an MOI of 0.01. Green signal represents dead cells stained by Cytotox Green Dye (#4633, Incucyte). Red signal represents the cells expressing mCherry-P2A-NLS-Cas13d.

Title: Supplementary Movie 4
Description: Time-lapse microscope imaging of MRC5 cells transduced with NLS-Cas13d and crRNA N20 and infected with 229E at an MOI of 0.01. Green signal represents dead cells stained by Cytotox Green Dye (#4633, Incucyte). Red signal represents the cells expressing mCherry-P2A-NLS-Cas13d.

Title: Supplementary Data 1
Description: crRNA sequences used in the study.

Title: Supplementary Data 2
Description: Primers and probes used for RT-qPCR in the study.

Title: Supplementary Data 3
Description: p values calculated in the study
